# Caryophyllene-type sesquiterpenes from the endophytic fungus *Pestalotiopsis lespedezae* through an OSMAC approach

**DOI:** 10.3389/fmicb.2023.1248896

**Published:** 2024-01-11

**Authors:** Xiaoqin Yu, Werner E. G. Müller, Marian Frank, Ying Gao, Zhiyong Guo, Kun Zou, Peter Proksch, Zhen Liu

**Affiliations:** ^1^Hubei Key Laboratory of Natural Products Research and Development, College of Biological and Pharmaceutical Sciences, China Three Gorges University, Yichang, China; ^2^Institute of Pharmaceutical Biology and Biotechnology, Heinrich-Heine-University Duesseldorf, Duesseldorf, Germany; ^3^Institute of Physiological Chemistry, Universitätsmedizin der Johannes Gutenberg-Universität Mainz, Mainz, Germany; ^4^Key Laboratory of Study and Discovery of Small Targeted Molecules of Hunan Province, School of Medicine, Hunan Normal University, Changsha, China

**Keywords:** *Pestalotiopsis lespedezae*, endophytic fungus, caryophyllene-type sesquiterpenes, pestalotiopsins, OSMAC approach, cytotoxicity

## Abstract

Two new caryophyllene-type sesquiterpenes pestalotiopsins U and V (**1** and **2**) and three known compounds pestalotiopsin B (**7**), pestaloporinate B (**8**), and pestalotiopsin C (**9**) were isolated by the cultivation of the endophytic fungus *Pestalotiopsis lespedezae* on solid rice medium, while four additional new caryophyllene pestalotiopsins W–Z (**3**–**6**) were obtained when 3.5% NaI was added to the fungal culture medium. The structures of the new compounds were determined by HRESIMS and 1D/2D nuclear magnetic resonance data. Compounds **1**–**9** were tested for cytotoxicity against the mouse lymphoma cell line L5178Y, but only **6** displayed significant activity with an IC_50_ value of 2.4 μM.

## Introduction

Fungi from the genus *Pestalotiopsis* are widely distributed in diverse substrata such as plants, soil, alga, polluted steam water, and extreme environments in temperate and tropical regions (Maharachchikumbura et al., 2011, 2014). They are famous not only as phytopathogens but also as endophytes living in a well-balanced equilibrium with their host plants (Hopkins and McQuilken, 2000). The discovery of new bioactive natural products from this genus has established it as a promising candidate for bioprospecting (Xu et al., 2010; Deshmukh et al., 2017). Examples of recently discovered new compounds from *Pestalotiopsis* include benzophenones from the marine-derived fungus *P. neglecta,* which were able to suppress cell proliferation and induce apoptosis of the pancreatic cancer cell line PANC-1 at a low micromolar dosage by targeting the MEK/ERK pathway (Wang et al., 2019). Cuautepestalorin, which was isolated from a bioactive extract of *Pestalotiopsis* sp., was found to be active when evaluated *in vitro* as an α-glucosidase inhibitor (Rivera-Chavez et al., 2019).

Caryophyllene is a sesquiterpene consisting of a four-membered ring and a flexible nine-membered ring with an *E*-configurated double bond. Caryophyllene is known as a constituent of many essential oils from plants (Rampogu et al., 2018), but it also occurs as a natural product of plant-associated fungi such as *Cytospora* spp. (Li et al., 2017), *Seiridium* spp. (Tayone et al., 2020), *Trichoderma* spp. (Yu et al., 2015), and *Pestalotiopsis* spp. (Liu S. et al., 2016; Liu Y. et al., 2016). Some caryophyllene derivatives have also been reported from marine sources. For instance, the caryophyllene-type sesquiterpenes punctaporonins H–M were obtained from the fermentation broth of the sponge-associated fungus *Hansfordia sinuosae,* while punctaporonin H was reported as a new natural product capable of reducing triglycerides and total cholesterol content (Wu et al., 2014). Pestalotiopsins A and B are highly oxygenated caryophyllene derivatives from *Pestalotiopsis* sp. isolated from *Taxus brevifolia* (Pulici et al., 1996). (+)-Pestalotiopsin A bears an unprecedented oxatricyclic core structure with seven stereogenic centers, and its absolute stereochemistry was established based on an enantioselective total synthesis of both enantiomers (Takao et al., 2008, 2009).

**Figure 1:**
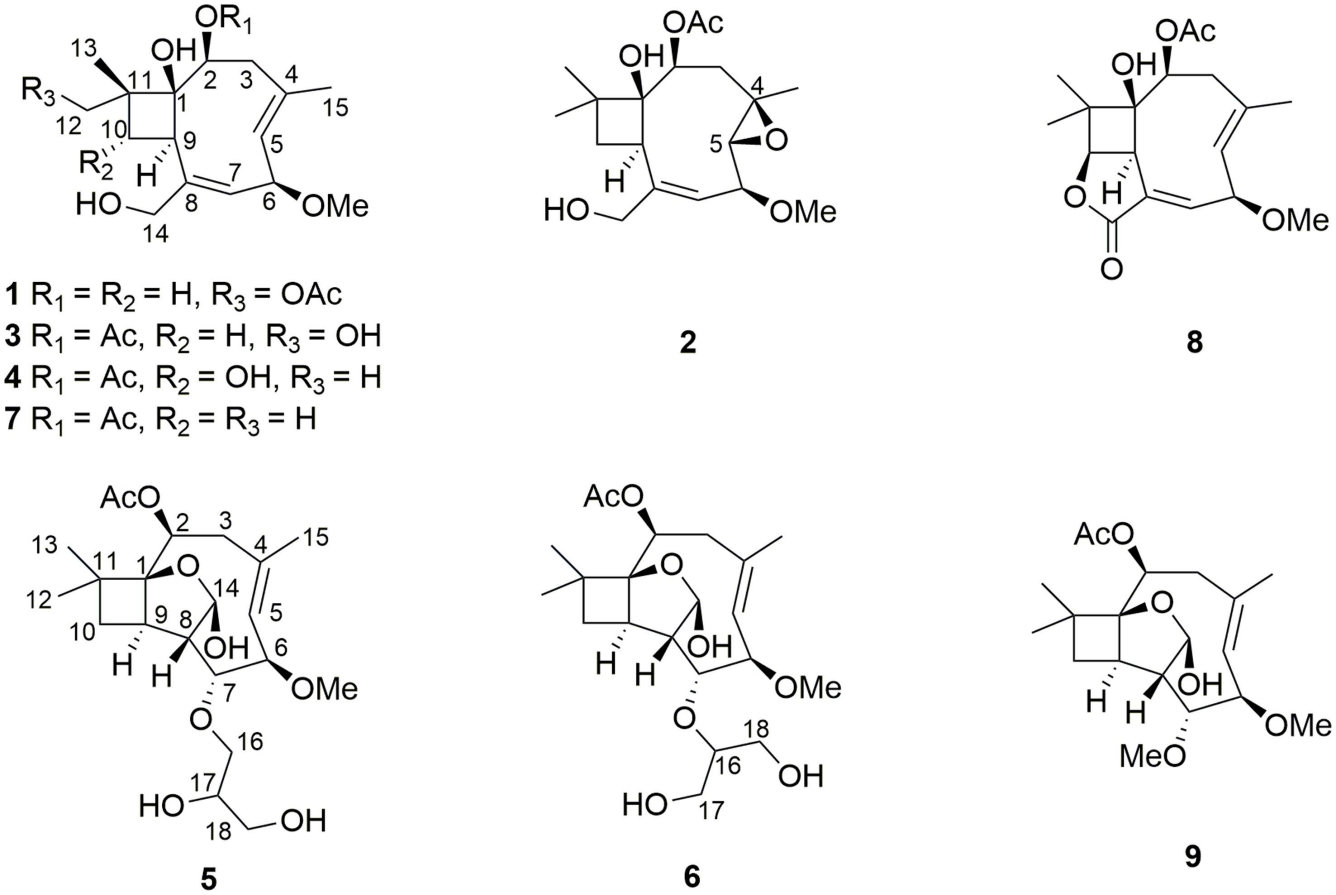
Structures of isolated compounds **1**–**9** from *P. lespedezae*.

The fungus *P. lespedezae* was isolated from the underground part of the medicinal plant *Youngia japonica* (L.) DC (Asteraceae) gathered in China. In a previous study, 10 new polyketide-terpenes, including a new iodized compound, were obtained following the addition of halogen salts such as NaCl, NaBr, and NaI to the solid rice medium (Yu et al., 2021). In the present study, we report two new caryophyllene-type sesquiterpenes pestalotiopsins U and V (**1** and **2**) and three known compounds pestalotiopsin B (**7**), pestaloporinate B (**8**), and pestalotiopsin C (**9**) from the solid rice medium culture of *P. lespedezae,* whereas four additional new caryophyllene pestalotiopsins W–Z (**3**–**6**) were obtained from the same fungus when cultured on solid rice medium with the addition of 3.5% NaI. All compounds were evaluated for their cytotoxicity against the mouse lymphoma cell line L5178Y, with only **6** showing significant cytotoxicity with an IC_50_ value of 2.4 μM (Figure 1).

## Materials and methods

### General experimental procedures

A Jasco P-2000 polarimeter was used for the measurement of optical rotations. Mass spectra (ESI) and HRESIMS data were obtained from an LC–MS HP1100 Agilent Finnigan LCQ Deca XP ThermoQuest spectrometer and a UHR-QTOF maXis 4G (Bruker Daltonics) mass spectrometer, respectively. NMR spectra were recorded with Bruker ARX 400 or 600 NMR spectrometers, with chemical shifts referring to the residual solvent peaks. Melting points were measured on an SGW X-4 micro-melting point instrument (Shanghai Jingke, China). A Dionex UltiMate-3400SD system (Thermo Scientific) coupled with an LPG-3400SD pump and a photodiode array detector (DAD 300RS) was employed for HPLC analysis. The analytical column (125 × 4 mm) was prefilled with Eurosphere-10 C18 (Knauer, Germany), and a gradient of MeOH and 0.1% formic acid in H_2_O was used: 0 min, 10% MeOH; 5 min, 10% MeOH; 35 min, 100% MeOH; 45 min, 100% MeOH. Semi-preparative HPLC was conducted using a Merck Hitachi HPLC System (UV detector L-7400; pump L-7100; Eurosphere-100 C_18_, 300 × 8 mm), and the mobile phase was a mixture of MeOH-H_2_O. Merck MN silica gel 60 M (0.04–0.063 mm) and Sephadex LH-20 were included in column chromatography. Thin-layer chromatography plates precoated with silica gel F_254_ were applied for monitoring and collecting fractions with detection under 254 and 365 nm or by spraying the plates with anise aldehyde reagent. The solvents used for column chromatography and spectroscopic measurements were distilled and spectral grade, respectively.

### Fungal material and identification

The fungus investigated in this study was obtained from the underground part of *Youngia japonica* (L.) DC (Asteraceae) collected by Prof. Han Xiao (Guizhou Nationalities University) from Guiyang, Guizhou, P.R. China, in April 2018. Based on the DNA amplification results and sequencing of its ITS region, this fungus was identified as *Pestalotiopsis lespedezae* (GenBank accession number MT521347). Details of the protocol are the same as described before (Kjer et al., 2010). A deep-frozen sample of this fungal strain (No. 8G-C) is deposited in the refrigerator at −80°C at the Institute of Pharmaceutical Biology and Biotechnology, Heinrich-Heine-University Duesseldorf.

### Fermentation, extraction, and isolation

Cultivation of *P*. *lespedezae* was conducted on solid rice medium in 10 Erlenmeyer flasks (1 L each). Each flask contained 100 g rice and 110 mL of demineralized water. The flasks were autoclaved at 121°C for 20 min before inoculation. After fermentation for 14 days at 20°C, 500 mL of EtOAc was added to each flask, followed by shaking the flasks at 140 rpm for 8 h. The crude extracts were obtained by filtration and removal of the solvent under reduced pressure.

In the OSMAC experiments, either 3.5 g NaCl, 3.5 g NaBr, or 3.5 g NaI were added to each flask containing 100 g rice and 110 mL of demineralized water. After fermentation and extraction, the chromatographic profiles of the respective extracts were obtained by HPLC. Based on the results, a large-scale fermentation was conducted by growing the fungus on the solid rice medium with the addition of 3.5 g NaI in 20 flasks.

The crude extract (8.5 g) from the rice fungal culture was dealt with a liquid–liquid partition between *n*-hexane and MeOH. The MeOH layer (4.6 g) was then subjected to vacuum liquid silica gel column chromatography with a gradient of *n*-hexane and EtOAc (100,1 to 0:100, v/v) as the mobile phase to give five fractions (Fr.1–5). Fr.2 (0.8 g) was then divided into three subfractions (Fr.2.1–2.3) by Sephadex LH-20 column chromatography (60 × 3 cm) using MeOH as eluent. Compounds **7** (10.0 mg, t_R_ = 20.6 min) and **8** (5.9 mg, t_R_ = 23.2 min) were yielded from Fr.2.2 (50 mg) through semi-preparative HPLC (MeOH-H_2_O: 0–3 min, 55%; 3–28 min, from 55 to 70%; 28–33 min, 100%), while compounds **1** (3.5 mg, t_R_ = 20.8 min), **2** (4.8 mg, t_R_ = 21.5 min), and **9** (32.5 mg, t_R_ = 24.0 min) were obtained from Fr.2.3 (100 mg) by semi-preparative HPLC (MeCN-H_2_O: 0–3 min, 15%; 3–23 min, from 15 to 25%; 23–31 min, 100%).

The large-scale fermentation of *P. lespedezae* on rice medium with the addition of 3.5 g NaI yielded 10.1 g of crude extract. In a similar manner, including liquid–liquid partition and vacuum liquid silica gel column chromatography as described above, four subfractions (Fr.1–4) were obtained. Fr.3 (2.0 g) was subjected to ODS column (60 × 200 mm) chromatography using MeOH to give five subfractions (Fr.3.1–3.5). Fr.3.3 (500 mg) was then submitted to a Sephadex LH-20 column (60 × 3 cm) with MeOH as eluent, followed by separation through semi-preparative HPLC (MeCN-H_2_O: 0–2 min, 15%; 2–25 min, from 15 to 20%; 25–30 min, 100%) to yield compounds **3** (4.5 mg, t_R_ = 14.4 min), **4** (3.7 mg, t_R_ = 16.2 min), **5** (2.0 mg, t_R_ = 23.3 min), and **6** (2.1 mg, t_R_ = 22.7 min).

Pestalotiopsin U (**1**): colorless oil, [*α*]20 D–55 (*c* 0.10, MeOH); UV (MeOH) λ_max_ 207 nm; ^1^H and ^13^C NMR data, see Table 1; HRESIMS *m/z* 363.1780 [M + Na] ^+^ (C_18_H_28_O_6_Na, calcd. 363.1778).

**Table 1:** NMR data for compounds **1** and **2**.

Position	Major conformer of**1** ^a^		Minor conformer of**1** ^a^		**2** ^b^	
	δ_C_, type	δ_H_ (*J* in Hz)	δ_C_, type	δ_H_ (*J* in Hz)	δ_C_, type	δ_H_ (*J* in Hz)
1	82.1, C		84.0, C		79.3, C	
2	73.1, CH	4.19, dd (11.0, 4.6)	76.2, CH	4.14, m	74.6, CH	5.29, dd (11.4, 4.2)
3	44.1, CH_2_	2.53, t (11.0) 2.33, dd (11.0, 4.6)	39.0, CH_2_	2.89, dd (14.5, 7.6) 1.80, m	38.8, CH_2_	2.33, dd (11.8, 4.2) 1.58, dd (11.8, 11.4)
4	133.2, C		142.4, C		56.4, C	
5	129.4, CH	5.13, d (10.2)	126.2, CH	5.62, d (7.3)	65.5, CH	2.93, d (9.2)
6	75.9, CH	4.46, dd (10.2, 1.9)	75.8, CH	4.27, dd (7.3, 1.0)	75.3, CH	3.80, dd (9.2, 1.6)
7	139.8, CH	5.94, d (1.9)	140.5, CH	5.98, d (1.0)	139.4, CH	5.96, d (1.6)
8	136.5, C		133.4, C		137.4, C	
9	40.7, CH	3.08, dd (10.4, 8.5)	45.6, CH	3.24, dd (11.0, 9.0)	40.9, CH	3.28, dd (10.8, 6.4)
10	29.2, CH_2_	2.00, dd (12.8, 8.5) 1.81, dd (12.8, 10.4)	31.6, CH_2_	2.09, dd (11.6, 11.0) 1.74, dd (11.6, 9.0)	32.9, CH_2_	1.99, dd (13.0, 6.4) 1.79, dd (13.0, 10.8)
11	44.5, C		43.8, C		42.7, C	
12	70.3, CH_2_	4.28, d (11.2) 4.17, d (11.2)	69.7, CH_2_	4.37, d (11.2) 4.27, d (11.2)	27.1, CH_3_	1.07, s
13	19.7, CH_3_	1.14, s	19.3, CH_3_	1.13, s	23.9, CH_3_	1.20, s
14	66.8, CH_2_	4.33, d (11.1) 3.93, d (11.1)	66.8, CH_2_	4.18, d (11.0) 3.92, d (11.0)	65.2, CH_2_	4.41, d (11.3) 4.03, d (11.3)
15	17.7, CH_3_	1.89, s	24.7, CH_3_	1.76, s	19.0, CH_3_	1.62, s
6-OMe	55.6, CH_3_	3.30, s	56.0, CH_3_	3.30, s	57.5, CH_3_	3.45, s
2-OAc					21.5, CH_3_ 170.1, C	2.06, s
12-OAc	21.1, CH_3_ 170.8, C	2.10, s	21.3, CH_3_ 171.0, C	2.00, s		

a Recorded at 400 MHz (^1^H) and 100 MHz (^13^C) in CDCl_3_.

b Recorded at 600 MHz (^1^H) and 150 MHz (^13^C) in CDCl_3_.

Pestalotiopsin V (**2**): needle crystals, m.p. 102–103°C, [*α*]20 D –64 (*c* 0.10, MeOH); UV (MeOH) λ_max_ 207 nm; ^1^H and ^13^C NMR data, see Table 1; HRESIMS *m/z* 363.1782 [M + Na] ^+^ (C_18_H_28_O_6_Na, calcd. 363.1778).

Pestalotiopsin W (**3**): colorless oil, [*α*]20 D–108 (*c* 0.30, MeOH); UV (MeOH) λ_max_ 207 nm; ^1^H and ^13^C NMR data, see Table 2; HRESIMS *m/z* 363.1780 [M + Na] ^+^ (C_18_H_28_O_6_Na, calcd. 363.1778).

**Table 2:** NMR data of compounds **3** and **4**.

Position	Major conformer of**3**^a^		Minor conformer of**3**^a^		Major conformer of**4** ^b^		Minor conformer of**4** ^b^	
	δ_C_, type	δ_H_ (*J* in Hz)	δ_C_, type	δ_H_ (*J* in Hz)	δ_C_, type	δ_H_ (*J* in Hz)	δ_C_, type	δ_H_ (*J* in Hz)
1	83.0, C		84.6, C		78.5, C		78.7, C	
2	76.9, CH	5.23, dd (10.8, 4.1)	80.0, CH	5.26, dd (10.5, 7.4)	79.4, CH	5.21, dd (10.5, 7.4)	76.2, CH	5.23, dd (10.7, 4.1)
3	41.2, CH_2_	2.59, t (10.8) 2.35, dd (10.8, 4.1)	36.3, CH_2_	2.95, dd (13.9, 7.4) 1.77, dd (13.9, 10.5)	35.2, CH_2_	3.01, dd (14.1, 7.4) 1.81, dd (14.1, 10.5)	40.2, CH_2_	2.55, t (10.7) 2.38, dd (10.7, 4.1)
4	134.1, C		142.7, C		142.0, C		132.6, C	
5	131.2, CH	5.15, d (10.1)	128.2, CH	5.84, d (7.0)	126.5, CH	5.73, d (7.1)	130.1, CH	5.15, d (10.0)
6	77.3, CH	4.56, d (10.1)	77.2, CH	4.37, d (7.0)	75.7, CH	4.34, d (7.1)	75.7, CH	4.49, d (10.0)
7	139.9, CH	5.94, br s	140.8, CH	5.96, br s	141.9, CH	6.08, br s	141.0, CH	6.02, br s
8	137.9, C		135.2, C		131.7, C		134.7, C	
9	41.0, CH	3.21, dd (10.5, 8.2)	46.6, CH	3.43, dd (11.1, 9.0)	56.0, CH	2.98, d (9.3)	51.9, CH	2.80, d (7.3)
10	30.0, CH_2_	2.14, dd (12.8, 8.2) 1.54, dd (12.8, 10.5)	32.7, CH_2_	2.28, t (11.1) 1.51, dd (11.1, 9.0)	73.9, CH	4.18, d (9.3)	73.7, CH	4.28, d (7.3)
11	46.6, C		45.8, C		46.1, C		45.7, C	
12	68.1, CH_2_	3.84, d (11.2) 3.42, d (11.2)	68.1, CH_2_	3.60, d (11.3) 3.51, d (11.3)	17.9, CH_3_	1.02, s	19.0, CH_3_	0.99, s
13	22.2, CH_3_	1.09, s	20.5, CH_3_	1.14, s	22.1, CH_3_	1.09, s	22.9, CH_3_	1.08, s
14	66.7, CH_2_	4.29, d (11.6) 3.93, d (11.6)	66.9, CH_2_	4.18, d (11.2) 3.89, d (11.2)	67.0, CH_2_	4.29, d (11.0) 4.02, d (11.0)	68.2, CH_2_	4.30, d (11.0) 4.00, d (11.0)
15	17.6, CH_3_	1.97, s	24.8, CH_3_	1.75, s	24.5, CH_3_	1.75, s	17.3, CH_3_	1.94, s
6-OMe	55.8, CH_3_	3.29, s	56.1, CH_3_	3.30, s	55.9, CH_3_	3.30, s	55.6, CH_3_	3.30, s
2-OAc	21.4, CH_3_ 172.0, C	2.03, s	21.4, CH_3_ 171.9, C	2.03, s	21.4, CH_3_ 170.0, C	2.06, s	21.4, CH_3_ 170.0, C	2.06, s

a Recorded at 600 MHz (^1^H) and 150 MHz (^13^C) in CD_3_OD.

b Recorded at 600 MHz (^1^H) and 150 MHz (^13^C) in CDCl_3_.

Pestalotiopsin X (**4**): colorless oil, [*α*]20 D–49 (*c* 0.10, MeOH); UV (MeOH) λ_max_ 207 nm; ^1^H and ^13^C NMR data, see Table 2; HRESIMS *m/z* 363.1781 [M + Na] ^+^ (C_18_H_28_O_6_Na, calcd. 363.1778).

Pestalotiopsin Y (**5**): colorless oil, [*α*]20 D–30 (*c* 0.15, MeOH); UV (MeOH) λ_max_ 207 nm; ^1^H and ^13^C NMR data, see Table 3; HRESIMS *m/z* 437.2137 [M + Na] ^+^ (C_21_H_34_O_8_Na, calcd. 437.2146).

**Table 3:** NMR data of compounds **5** and **6**.

Position	**5** ^a^		**6** ^a^	
	δ_C_, type	δ_H_ (*J* in Hz)	δ_C_, type	δ_H_ (*J* in Hz)
1	99.2, C		98.7, C	
2	74.9, CH	5.25, dd (10.6, 5.5)	75.0, CH	5.24, dd (10.7, 5.4)
3	42.0, CH_2_	2.48, dd (10.6, 5.5) 2.46, t (10.6)	42.2, CH_2_	2.50, dd (10.7, 5.4) 2.42, t (10.7)
4	138.2, C		138.0, C	
5	125.9, CH	5.15, d (11.6)	126.0, CH	5.15, d (11.8)
6	84.5, CH	3.88, dd (11.6, 5.8)	83.3, CH	4.05, dd (11.8, 5.7)
7	78.6, CH	3.93, dd (5.8, 2.2)	87.4, CH	3.63, dd (5.7, 2.3)
8	66.5, CH	2.46, ddd (3.1, 2.2, 1.5)	64.4, C	2.45, ddd (2.7, 2.3, 1.5)
9	39.2, CH	2.64, ddd (9.7, 6.2, 1.5)	39.6, CH	2.62, ddd (9.7, 6.2, 1.5)
10	43.4, CH_2_	1.98, dd (12.0, 9.7) 1.56, dd (12.0, 6.2)	43.4, CH_2_	1.97, dd (11.9, 9.7) 1.58, dd (11.9, 6.2)
11	40.8, C		40.6, C	
12	27.5, CH_3_	1.03, s	27.7, CH_3_	1.02, s
13	24.8, CH_3_	1.11, s	24.7, CH_3_	1.17, s
14	116.5, CH	5.46, d (3.1)	108.9, CH	5.78, d (2.7)
15	17.6, CH_3_	1.92, s	17.5, CH_3_	1.91, s
16	71.3, CH_2_	3.90, m 3.60, m	72.4, CH	3.72, m
17	72.4, CH	3.83, m	72.3, CH_2_	3.60, m 3.52, m
18	64.7, CH_2_	3.62, m 3.53, m	64.5, CH_2_	3.57, m 3.51, m
6-OMe	56.5, CH_3_	3.29, s	56.2, CH_3_	3.28, s
2-OAc	21.3, CH_3_ 172.2, C	2.02, s	21.3, CH_3_ 172.2, C	2.01, s

a Recorded at 600 MHz (^1^H) and 150 MHz (^13^C) in CD_3_OD.

Pestalotiopsin Z (**6**): colorless oil, [*α*]20 D–18 (*c* 0.18, MeOH); UV (MeOH) λ_max_ 207 nm; ^1^H and ^13^C NMR data, see Table 3; HRESIMS *m/z* 437.2142 [M + Na] ^+^ (C_21_H_34_O_8_Na, calcd. 437.2146).

### Cytotoxicity assay

MTT method was applied to evaluate the cytotoxicity of all isolated compounds against the mouse lymphoma cell line L5178Y. Kahalalide F and 0.1% EGMME/DMSO were used as positive and negative controls, respectively (Ashour et al., 2006). The cells were grown in RPMI medium and 10% fetal calf serum (FCS) (Biochrom/Merck). Thiazolyl blue tetrazolium bromide (MTT; # M2128 Sigma) was used as an indicator for cell viability. The viability assay reaction is based on the oxidation of the yellowish MTT solution to solid formazan via mitochondrial dehydrogenases in living cells. The crystals formed were solubilized with acidified isopropanol, and the intensity was determined colorimetrically at 570 nm (Chuenjitkuntaworn et al., 2016).

## Results and discussion

**Figure 2:**
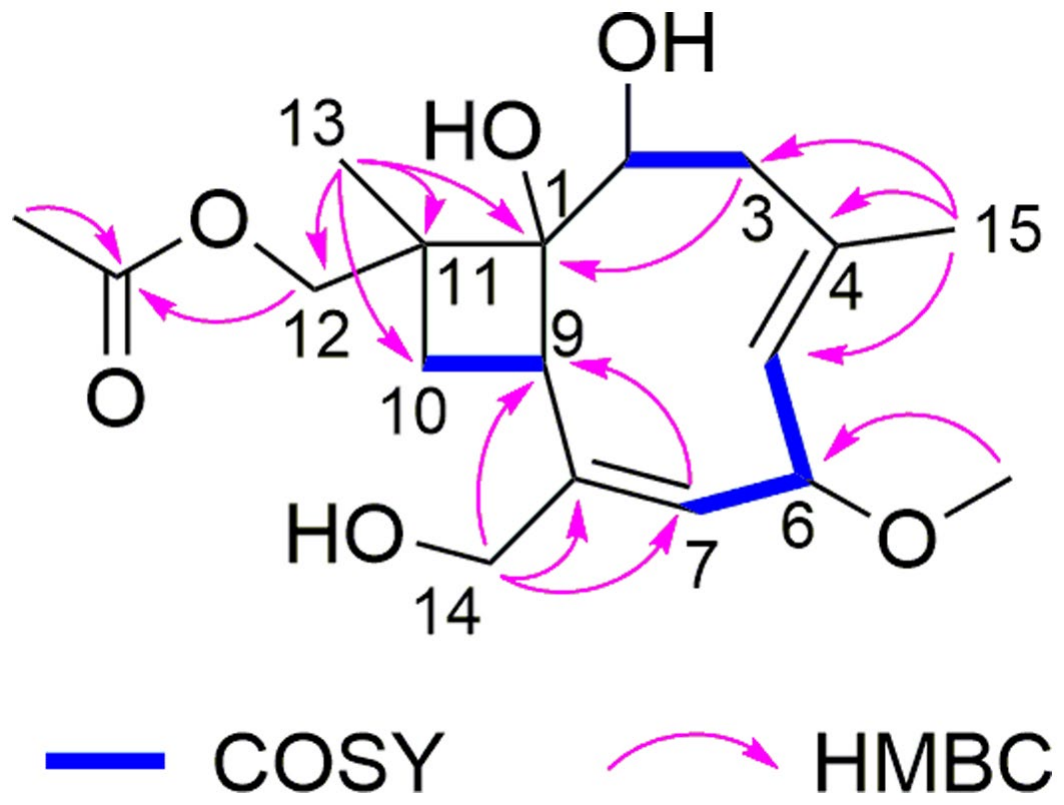
COSY and key HMBC correlations for compound **1**.

**Figure 3:**
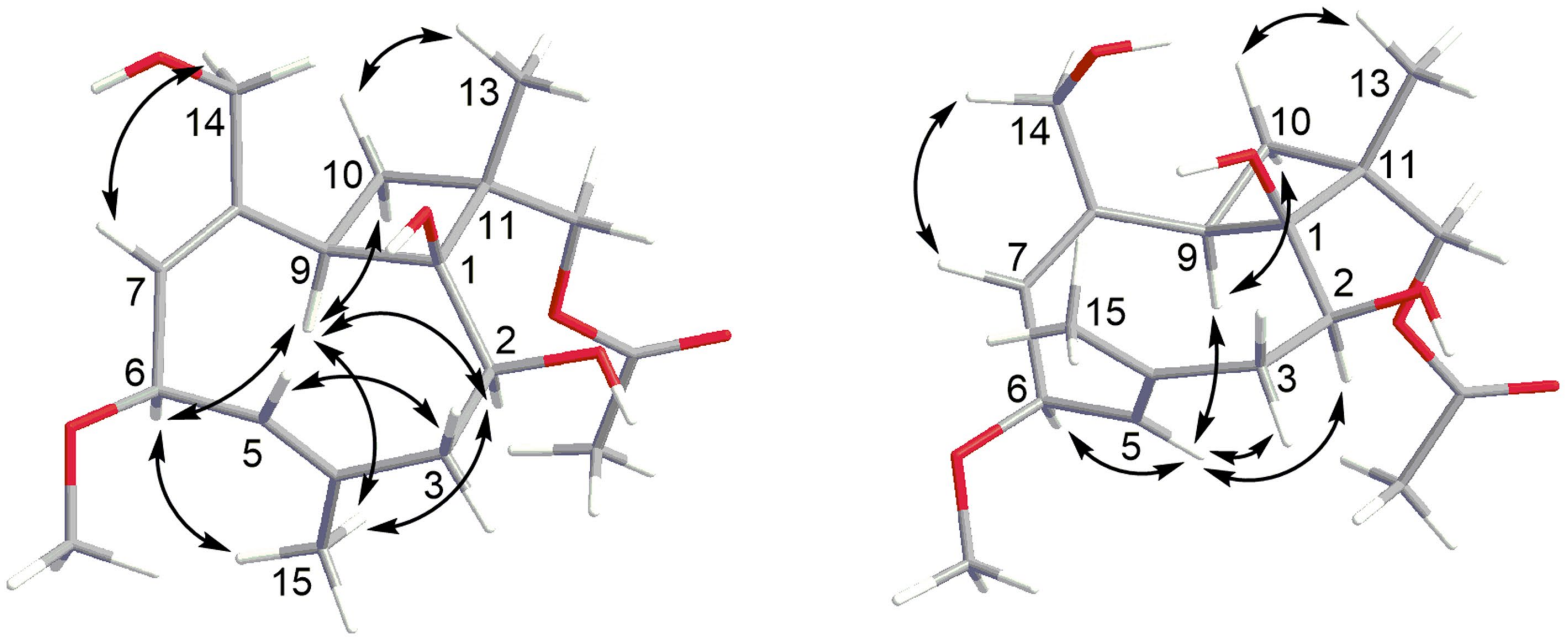
Key NOE correlations for major (left) and minor (right) conformers of compound **1**.

The HRESIMS data of **1** showed the pseudomolecular ion peaks at *m*/*z* 341.1960 [M + H]^+^, 358.2225 [M + NH_4_]^+^, and 363.1780 [M + Na]^+^, which indicated the molecular formula C_18_H_28_O_6_ with five degrees of unsaturation, containing one additional oxygen atom by comparison with that of the co-isolated known compound pestalotiopsin B (**7**) (Pulici et al., 1996). In the ^1^H NMR spectrum, compound **1** was observed as a mixture of two conformational isomers with a ratio of approximately 80:20, which was also found for pestalotiopsin B (**7**) and other previously reported caryophyllene-type sesquiterpenes such as pestalotiopsins C_1_ (Yu et al., 2015), M, and N (Tayone et al., 2020). The ^1^H and ^13^C NMR data of **1** (Table 1) resembled those of pestalotiopsin B (**7**). However, signals of the one methyl group were replaced by signals of an oxygenated methylene at *δ*_C_ 70.3, *δ*_H_ 4.28 and 4.17 (CH_2_-12, major conformer) in **1**. The HMBC correlations from Me-13 (*δ*_H_ 1.14) to C-1 (*δ*_C_ 82.1), C-10 (*δ*_C_ 29.2), C-11 (*δ*_C_ 44.5), and C-12, as well as the HMBC correlations from H_2_-12 and protons of the acetoxy group (*δ*_H_ 2.10) to the carbonyl carbon (*δ*_C_ 170.8) of the acetoxy group, confirmed the location of the acetoxy group at C-12 in **1** rather than location at C-2 in pestalotiopsin B (**7**). The remaining structure of **1** was found to be identical to that of **7** after the detailed analysis of the 2D NMR spectra of **1** (Figure 2). 4*E* and 7*E* geometries were assigned by NOE correlations of H-5/H-3a, H-7/H_2_-14 in both conformers of **1** (Figure 3). However, characteristic differences between the two conformers were found for the ROESY correlations associated with H-5 and Me-15. In the major conformer of **1**, the ROESY correlations between Me-15/H-2, Me-15/H-6, Me-15/H-9, H-9/H-2, H-9/H-6, H-9/H-10b (*δ*_H_ 1.81), and between Me-13/H-10a (*δ*_H_ 2.00) established that H-2, H-6, H-9, and Me-15 were on the same face of the ring, whereas OH-1 and Me-13 were on the opposite side. In the minor conformer, the ROESY correlations from H-5 to H-2, H-6 and H-9, and between H-9/H-10b (*δ*_H_ 1.74), Me-13/H-10a (*δ*_H_ 2.09) indicated α-orientation of H-2, H-5, H-6, and H-9, whereas OH-1 and Me-13 were β-oriented. Thus, the relative configuration of **1** was assigned as shown, and the obvious difference in the two conformational isomers was the orientation of H-5 and Me-15 caused by coupled hindered rotations regarding the C-3/C-4 and C-5/C-6 bonds. Thus, compound **1** was elucidated as a new caryophyllene derivative, for which the trivial name pestalotiopsin U is proposed.

Compound **2** had the molecular formula C_18_H_28_O_6_ as established by the sodiated-molecular peak at *m/z* 363.1782 in the HRESIMS data, containing one additional oxygen atom when compared to that of pestalotiopsin B (**7**) (Pulici et al., 1996). The ^1^H and ^13^C NMR data (Table 1) of **2** were similar to those of **7,** except for the fact that two olefinic carbons were replaced by two oxygenated carbons at *δ*_C_ 65.5 (C-5) and 56.4 (C-4) in **2**. An epoxy substituent at C-4/C-5 was evident from the COSY correlations between H-5 (*δ*_H_ 2.93)/H-6 (*δ*_H_ 3.80)/H-7 (*δ*_H_ 5.96), together with the HMBC correlations from Me-15 (*δ*_H_ 1.62) to C-3 (*δ*_C_ 38.8), C-4, and C-5. Detailed analysis of the 2D NMR spectra for **2** revealed that the remaining planar structure of **2** was identical to that of pestalotiopsin B (**7**). The double bond of C-7/C-8 was *E*-configurated as determined by the NOE correlation between H-7 and H-14b (*δ*_H_ 4.03). Moreover, the ROESY correlations between H-2 (*δ*_H_ 5.29)/Me-15, Me-15/H-6, H-6/H-9 (*δ*_H_ 3.28), H-9/H-2, H-2/H-3a (*δ*_H_ 2.33), H-3a/Me-15, and Me-15/H-9 indicated the cofacial orientation of these protons, and thus the relative configuration of **2** was determined as shown.

Pestalotiopsin W (**3**) possessed the same molecular formula as **1**, according to the sodiated-molecular peak at *m/z* 363.1780 in the HRESIMS data. The ^1^H NMR spectrum of **3** (Table 2) suggested an approximately 56:44 mixture of two conformers. Comparison of the ^1^H and ^13^C NMR data of **3** and **1** suggested that their structures were closely related. The major differences found were a downfield shifted oxygenated methine (H-2, *δ*_H_ 5.23 in major conf. and 5.26 in minor conf. in **3** compared to *δ*_H_ 4.19 in major conf. and 4.14 in minor conf. in **1**) together with an upfield shifted oxygenated methylene (H_ab_-12, *δ*_H_ 3.84, 3.42 in major conf. and 3.60, 3.51 in minor conf. in **3** compared to *δ*_H_ 4.28, 4.17 in major conf. and 4.37, 4.27 in minor conf. in **1**). These findings suggested the attachment of the acetoxy group at C-2 in **3** rather than at C-12 in **1**, which was further confirmed by the COSY correlation between H_ab_-3 and the above-mentioned downfield-shifted oxygenated methine together with the HMBC correlation from the latter and a methyl group at *δ*_H_ 2.03 to the carbonyl carbon *δ*_C_ 172.0 or 171.9 in both conformers of **3**. Detailed analysis of the 2D NMR spectra of **3** revealed that the remaining substructure and relative configuration of **3** were identical to those of **1**.

Based on the HRESIMS data, the molecular formula of **4** was deduced as C_18_H_28_O_6_, which was identical to that of **3**. Two sets of signals with a ratio of approximately 73:27 were observed in the ^1^H NMR spectrum of **4** (Table 2). Analysis of the ^1^H and ^13^C NMR data (Table 3) revealed structural similarity between **4** and **3**. However, the replacement of signals for a hydroxymethyl group by those of an additional methyl group (Me-12) at *δ*_H_ 1.02 and *δ*_C_ 17.9 (major conf.) or at *δ*_H_ 0.99 and *δ*_C_ 19.0 (minor conf.) in **4** indicated a different location of the hydroxy group in **4**. The COSY correlation between H-10 (*δ*_H_ 4.18 and 4.28 for major and minor conformers, respectively) and H-9, together with the HMBC correlations from the additional methyl group Me-12 to C-1, C-10, C-11, and C-13, confirmed the location of a hydroxy group at C-10 in **4** rather than at C-12 in **3**. The remaining structure of **4** was determined to be identical to that of **3** after the detailed analysis of the 2D NMR spectra of **4**. The olefinic double bonds at C-4/C-5 and C-7/C-8 were shown to be *E*-configurated, as indicated by the NOE correlations between H-5/H-3a and H-7/H_2_-14 in both conformers of **4**, respectively. In the major conformer of **4**, the NOE correlations between H-6/H-5, H-5/H-9, H-9/Me-12, Me-12/H-2, H-2/H-5, and between Me-13 and H-10 indicated *α*-orientation of H-2, H-5, H-6, H-9, and Me-12, whereas OH-1, Me-13, and H-10 were *β*-orientated. Meanwhile, in the minor conformer of **4**, the NOE correlations between H-6/Me-15, Me-15/H-9, H-9/Me-12, Me-12/H-2, H-2/Me-15, and between Me-13 and H-10 indicated the *α*-orientation of H-2, H-6, H-9, Me-12, and Me-15, whereas OH-1, Me-13, and H-10 were *β*-orientated. Thus, the relative configuration at C-1, C-2, C-6, and C-9 of **4** was assigned to be identical as **1** and **3**, while the additional OH-10 was α-oriented in **4**. It is worth mentioning that in **1** and **3**, H-5 and H-9 were aligned on the opposite and the same side of the cyclononadiene ring in the major and minor conformers, respectively, whereas in **4**, H-5 and H-9 were aligned on the same and the opposite side of the cyclononadiene ring in the major and minor conformers, respectively.

**Figure 4:**
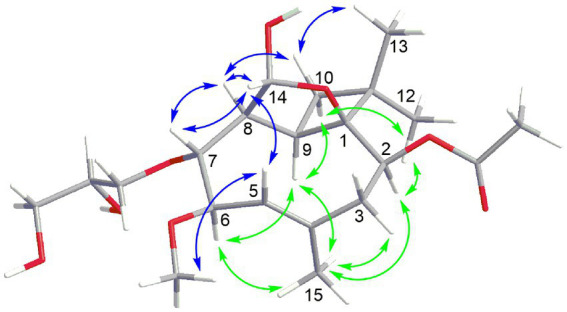
Key NOE correlations for compound **5**.

Compound **5** had the molecular formula C_21_H_34_O_8_ as deduced by HRESIMS, indicating 5 degrees of unsaturation. The ^13^C NMR data of **5** (Table 3) displayed a carbonyl carbon at *δ*_C_ 172.2 and carbon signals of a double bond at *δ*_C_ 138.2 and 125.9, accounting for two of the five degrees of unsaturation, thus implying the presence of a tricyclic ring system. The ^1^H and ^13^C NMR data of **5** were almost identical to those of co-isolated known tricyclic caryophyllene pestalotiopsin C (**9**) (Pulici et al., 1997), except for the absence of signals for a methoxy group and appearance of two additional oxygenated methylene (CH_2_-16 at *δ*_C_ 71.3, *δ*_H_ 3.90 and 3.60; CH_2_-18 at *δ*_C_ 64.7, *δ*_H_ 3.62 and 3.53) and an additional oxygenated methine (CH-17 at *δ*_C_ 72.4, *δ*_H_ 3.83) in **5**. The COSY correlations between H-5/H-6/H-7/H-8/H-14 and between H_ab_-16/H-17/H_ab_-18, together with the HMBC correlations from H_ab_-16 to C-7, indicated the attachment of an additional 2,3-dihydroxypropoxy group at C-7. The remaining structure of **5** was determined to be identical to that of **9** after detailed analysis of the 2D NMR data of **5**. In the ROESY spectrum (Figure 4), the cross-peaks between Me-13/H-10b (*δ*_H_ 1.56), H-10b/H-8, H-8/H-7, H-7/H-14, H-14/H-8, H-14/H-5, H-5/OMe-6, H-5/H-3b (*δ*_H_ 2.46), and between H-9/H-10a (*δ*_H_ 1.98), H-10a/Me-12, Me-12/H-2, H-2/Me-15, Me-15/H-6, H-6/H-9, H-9/Me-15, and Me-15/H-3a (*δ*_H_ 2.48) indicated the relative configuration of **5** as shown, which is identical to that of structurally related pestalotiopsins A (Pulici et al., 1996), C (9) (Pulici et al., 1997), E (Putra et al., 2019) and J (Xiao et al., 2017).

The molecular formula of pestalotiopsin Z (**6**) was determined to be identical to that of **5** based on the HRESIMS data. Comparison of their ^1^H and ^13^C NMR data (Table 2) and analysis of the 2D NMR spectra indicated that both compounds shared the same tricyclic caryophyllene core in addition to the presence of a glycerol subunit. However, the HMBC correlation from the oxygenated methine at *δ*_H_ 3.72 (H-16) in **6** instead of an oxygenated methylene in **5** to C-7 confirmed that the substituent at C-7 was (1,3-dihydroxypropan-2-yl)oxy in **6** rather than 2,3-dihydroxypropoxy in **5**. The relative configuration of **6** was assigned to be identical to that of **5** by the comparison of their coupling constants and NOE relationships.

Sequencing data of the fungal genomes indicated that a large part of secondary metabolic gene clusters are silent under standard laboratory culture conditions (Debbab et al., 2013; Rutledge and Challis, 2015). In our current research, more new analogs were isolated when 3.5% NaI was added. These results, together with other reported successful examples (Wang et al., 2015; Yamazaki et al., 2015), suggested that OSMAC could be an effective approach that may result in a steep accumulation of target compounds or enhancement of diverse secondary metabolites from fungi by activating their silent gene clusters (Wiemann and Keller, 2014).

Pestalotiopsins A-T have been reported from different fungi so far (Pulici et al., 1996, 1997; Baker et al., 2008; Xiao et al., 2017; Putra et al., 2019; Tayone et al., 2020; Kang et al., 2023); however, they seldom showed cytotoxicity. In our study, all compounds were tested for cytotoxicity against the mouse lymphoma cell line L5178Y, but only compound **6** exhibited potent cytotoxicity with an IC_50_ value of 2.4 μM compared to the positive control of 4.3 μM. The other tested compounds did not show any inhibitory effect at a concentration of 10 μM. The presence of a (1,3-dihydroxypropan-2-yl)oxy moiety in **6** rather than 2,3-dihydroxypropoxy in **5** or a methoxy group in **9** might contribute to the cytotoxicity.

## Conclusion

In summary, six new caryophyllene-type sesquiterpenes pestalotiopsins U–Z (**1**–**6**) and three known derivatives pestalotiopsin B (**7**), pestaloporinate B (**8**), and pestalotiopsin C (**9**) were obtained through the fermentation of the endophytic fungus *P*. *lespedezae* on solid rice medium. Pestalotiopsins W–Z (**3**–**6**) were not detected when the fungus was grown on rice medium without the addition of 3.5% NaI. Cytotoxicity against the mouse lymphoma cell line L5178Y of all isolated compounds was evaluated, but only pestalotiopsin Z (**6**) showed significant activity with an IC_50_ value of 2.4 μM.

## Data availability statement

The datasets presented in this study can be found in online repositories. The names of the repository/repositories and accession number(s) can be found in the article/Supplementary material.

## Author contributions

XY contributed to extraction, isolation, and manuscript preparation. WM carried out cytotoxicity assay. MF and YG contributed to part of fungal fermentation. ZG and KZ contributed to part of structure elucidation. PP and ZL supervised the research work and revised the manuscript. All authors contributed to the article and approved the submitted version.
