# Supplementary material for: Caryophyllene-type sesquiterpenes from the endophytic fungus Pestalotiopsis lespedezae through an OSMAC approach
Source: Front Microbiol. 2024 Jan 11;14:1248896. doi: 10.3389/fmicb.2023.1248896 (PMC10808731; doi:10.3389/fmicb.2023.1248896)
Supplement: Supplementary file 1 [file Data_Sheet_1.PDF]

Supporting Information

Caryophyllene-type Sesquiterpenes from the Endophytic Fungus *Pestalotiopsis lespedezae* through an OSMAC Approach

Xiaoqin Yu[1, 2], Werner E.G. Müller[3], Marian Frank[2], Ying Gao[2], Zhiyong Guo[1], Kun Zou[1], Peter Proksch[2]*, Zhen Liu[4]*

[1] Hubei Key Laboratory of Natural Products Research and Development, College of Biological and Pharmaceutical Sciences, China Three Gorges University, Yichang, China,

[2] Institute of Pharmaceutical Biology and Biotechnology, Heinrich-Heine-University Duesseldorf, Duesseldorf, Germany,

[3] Institute of Physiological Chemistry, Universitätsmedizin der Johannes Gutenberg-Universität Mainz, Mainz, Germany,

[4] Key Laboratory of Study and Discovery of Small Targeted Molecules of Hunan Province, School of Medicine, Hunan Normal University, Changsha, China

*****Correspondence:

Peter Proksch

proksch@uni-duesseldorf

Zhen Liu

liuzhen2020@hunnu.edu.cn

Table of Contents

Figure S1. The HRESIMS of compound **1**……………….…….…………………………4
Figure S2. The UV spectrum of compound **1**…………………………………………4
Figure S3. The ^{1}H-NMR spectrum of compound **1**………………..…………………5
Figure S4. The ^{13}C-NMR spectrum of compound **1**……………………………….5
Figure S5. The ^{1}H-^{1}H COSY spectrum of compound **1**……………………………6
Figure S6. The HSQC spectrum of compound **1**……………………………..……6
Figure S7. The HMBC spectrum of compound **1**……………………….…………7
Figure S8. The ROESY spectrum of compound **1**…………………………………7
Figure S9. The HRESIMS of compound **2**…………………………………………8
Figure S10. The UV spectrum of compound **2**……………………………….………8
Figure S11. The ^{1}H-NMR spectrum of compound **2**……………………………...…9
Figure S12. The ^{13}C-NMR spectrum of compound **2**……………………………...…9
Figure S13. The ^{1}H-^{1}H COSY spectrum of compound **2**…………………………10
Figure S14. The HSQC spectrum of compound **2**……………………………...…10
Figure S15. The HMBC spectrum of compound **2**……………………………..…11
Figure S16. The ROESY spectrum of compound **2**………………………………11
Figure S17. The HRESIMS of compound **3**…………………………….……….12
Figure S18. The UV spectrum of compound **3**……………………………………12
Figure S19. The ^{1}H-NMR spectrum of compound **3**…………………….……….13
Figure S20. The ^{13}C-NMR spectrum of compound **3**……………………………..…13
Figure S21. The ^{1}H-^{1}H COSY spectrum of compound **3**…………………………14
Figure S22. The HSQC spectrum of compound **3**……………………………...…14
Figure S23. The HMBC spectrum of compound **3**………………………….…….15
Figure S24. The ROESY spectrum of compound **3**………………………………15
Figure S25. The HRESIMS of compound **4**…………....……………………………16
Figure S26. The UV spectrum of compound **4**……………………………………...…16
Figure S27. The ^{1}H-NMR spectrum of compound **4**……………………………….17
Figure S28. The ^{13}C-NMR spectrum of compound **4**……………………………..…17
Figure S29. The ^{1}H-^{1}H COSY spectrum of compound **4**…………………………18
Figure S30. The HSQC spectrum of compound **4**……………………………...…18
Figure S31. The HMBC spectrum of compound **4**…..……………………….……19
Figure S32. The ROESY spectrum of compound **4**………………………………19
Figure S33. The HRESIMS of compound **5**………………….………………………20
Figure S34. The UV spectrum of compound **5**……………………………………20
Figure S35. The ^{1}H-NMR spectrum of compound **5**……………………………...…20
Figure S36. The ^{1}H-^{1}H COSY spectrum of compound **5**…………………………21
Figure S37. The HSQC spectrum of compound **5**……………………………...…21
Figure S38. The HMBC spectrum of compound **5**……………………………….22
Figure S39. The ROESY spectrum of compound **5**………………………………22
Figure S40. The HRESIMS of compound **6**………..……………………………...23
Figure S41. The UV spectrum of compound **6**……………………………………24
Figure S42. The ^{1}H-NMR spectrum of compound **6**……………………………...24

Figure S43. The ^{1}H-^{1}H COSY spectrum of compound **6**……………………………25
Figure S44. The HSQC spectrum of compound **6**…………………………………...25
Figure S45. The HMBC spectrum of compound **6**………………………..………26
Figure S46. The ROESY spectrum of compound **6**…………………………………26

Acquisition Parameter

Source Type	ESI	Ion Polarity	Positive	Set Nebulizer	0.3 Bar
Focus	Not active	Set Capillary	4000 V	Set Dry Heater	180 °C
Scan Begin	50 m/z	Set End Plate Offset	-500 V	Set Dry Gas	4.0 l/min
Scan End	1500 m/z	Set Collision Cell RF	600.0 Vpp	Set Divert Valve	Source

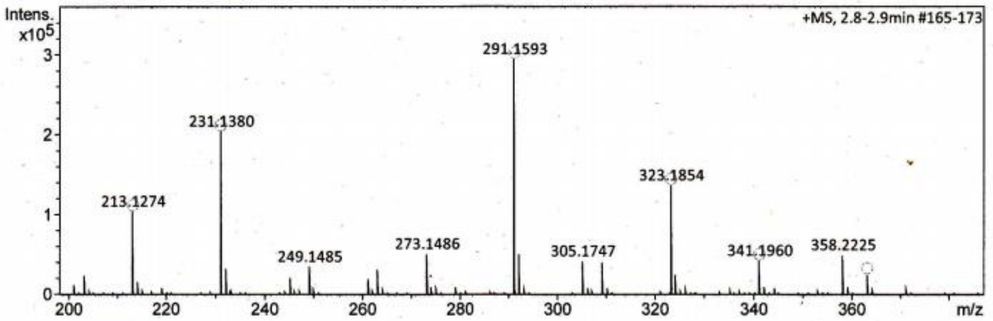

Meas. m/z	#	Ion Formula	m/z	err [ppm]	mSigma	# mSigma	Score	rdb	e⁻ Conf	N-Rule
213.1274	1	C15H17O	213.1274	0.1	16.3	1	100.00	7.5	even	ok
231.1380	1	C15H19O2	231.1380	-0.4	5.2	1	100.00	6.5	even	ok
291.1593	1	C17H23O4	291.1591	-0.7	10.0	1	100.00	6.5	even	ok
323.1854	1	C18H27O5	323.1853	-0.2	8.8	1	100.00	5.5	even	ok
341.1960	1	C18H29O6	341.1959	-0.3	4.1	1	100.00	4.5	even	ok
363.1780	1	C18H28NaO6	363.1778	-0.5	5.2	1	100.00	4.5	even	ok

Figure S1. The HRESIMS of compound **1**.

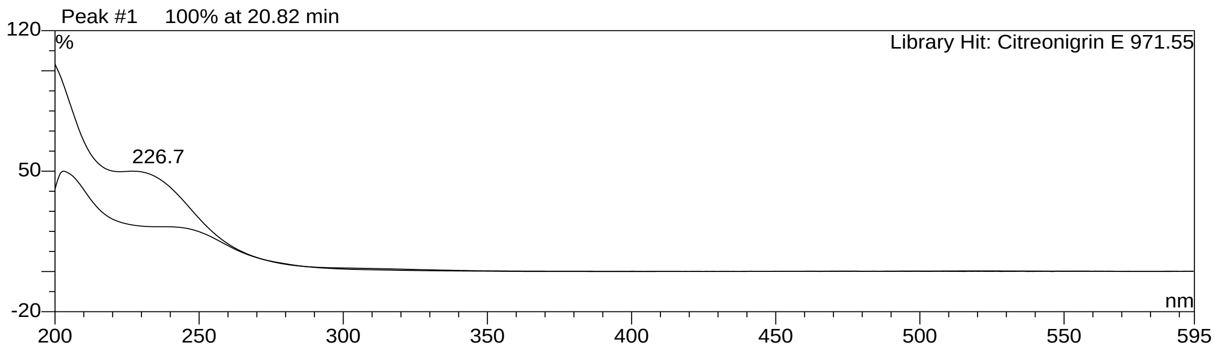

Figure S2. The UV spectrum of compound **1**.

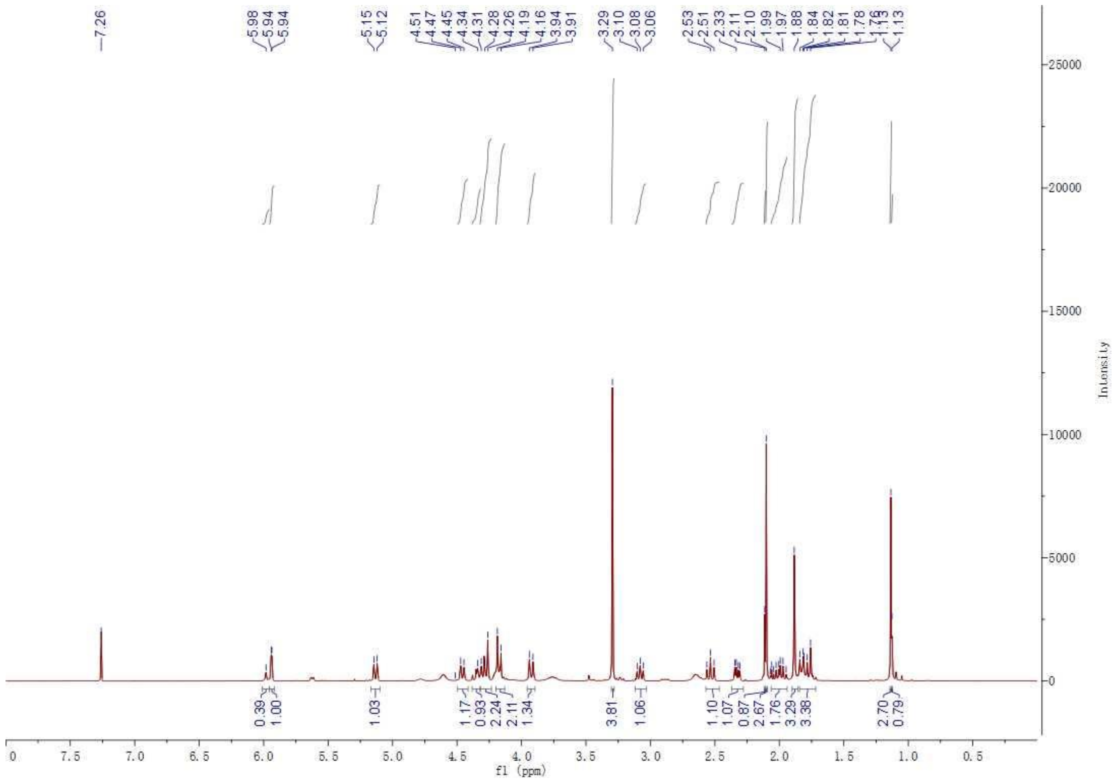

Figure S3. The ^{1}H-NMR (400 MHz, $CDCl_3$) spectrum of compound **1**.

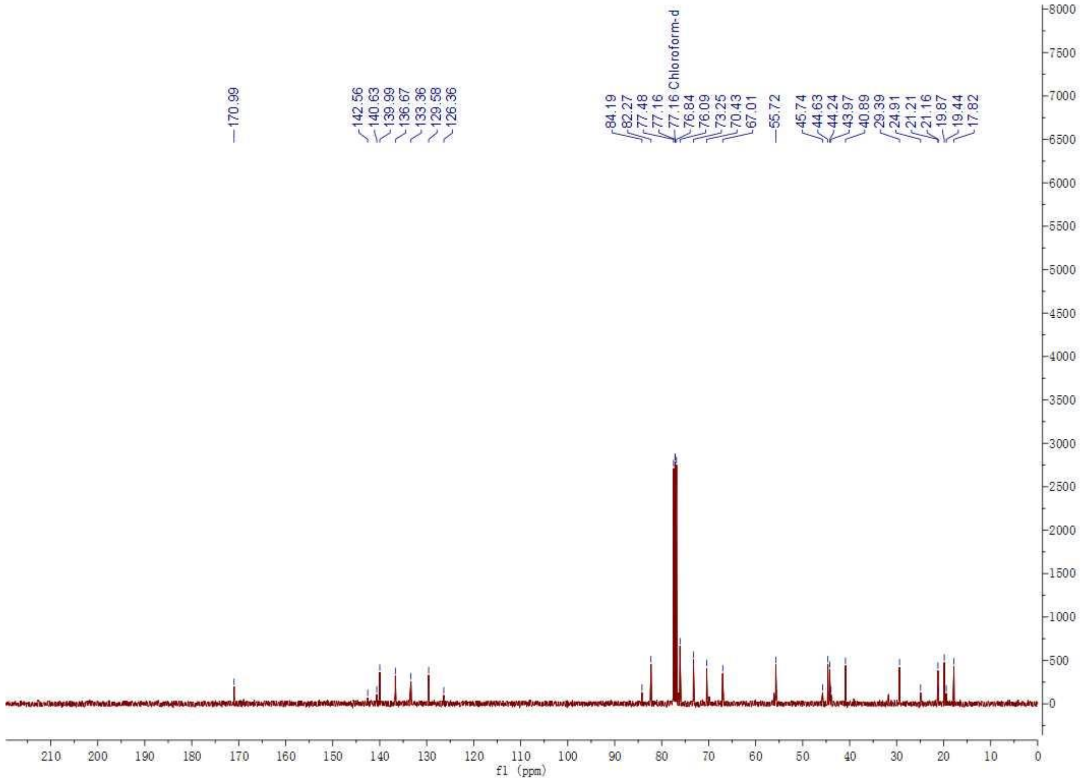

Figure S4. The ^{13}C-NMR (100 MHz, $CDCl_3$) spectrum of compound **1**.

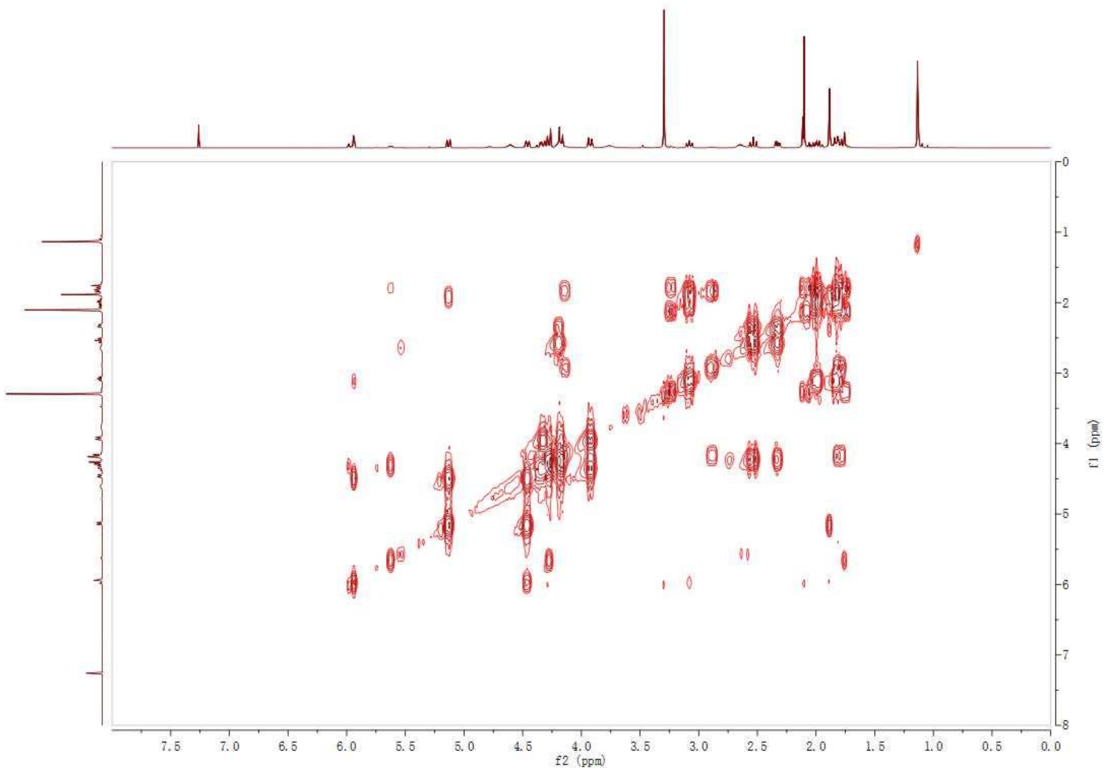

Figure S5. The ^{1}H-^{1}H COSY (400 MHz, $CDCl_3$) spectrum of compound **1**.

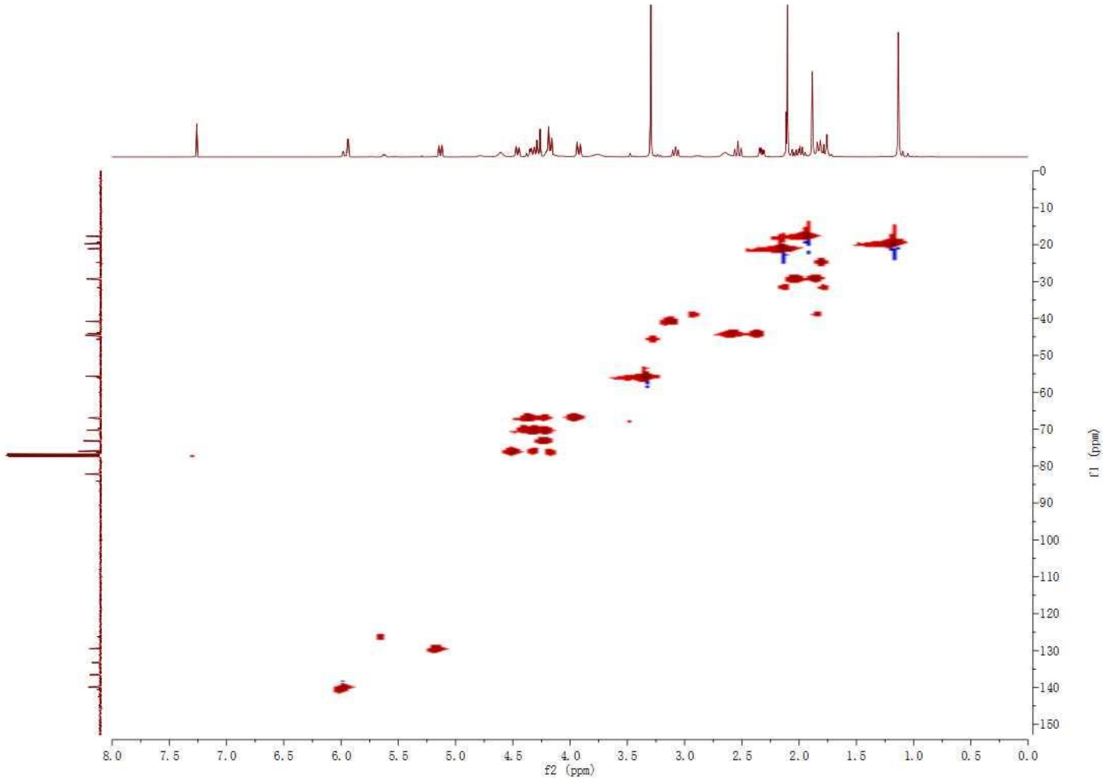

Figure S6. The HSQC (400 MHz, $CDCl_3$) spectrum of compound **1**.

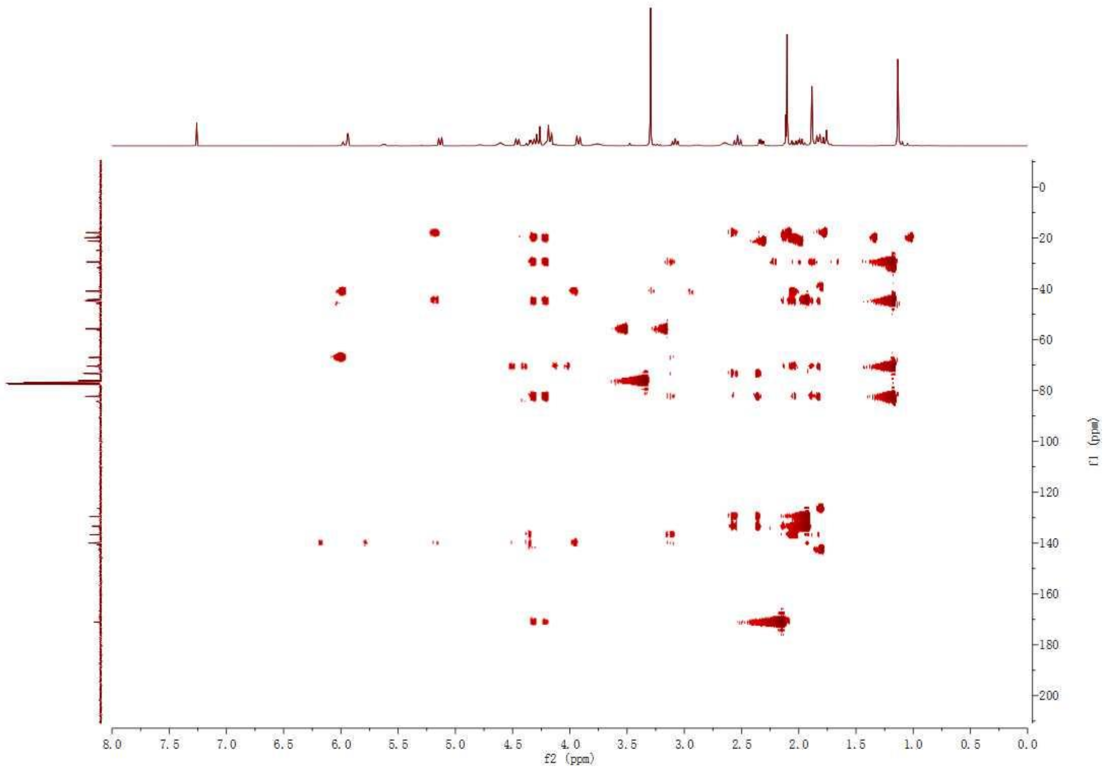

Figure S7. The HMBC (400 MHz, $CDCl_3$) spectrum of compound **1**.

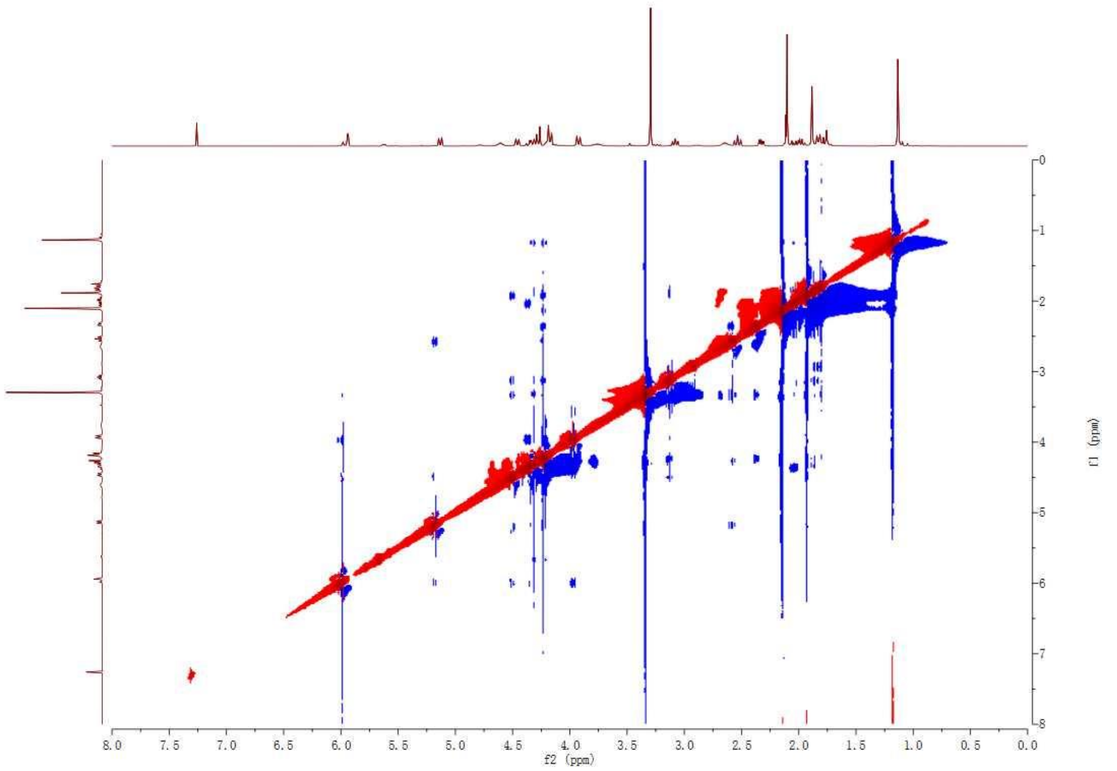

Figure S8. The ROESY (400 MHz, $CDCl_3$) spectrum of compound **1**.

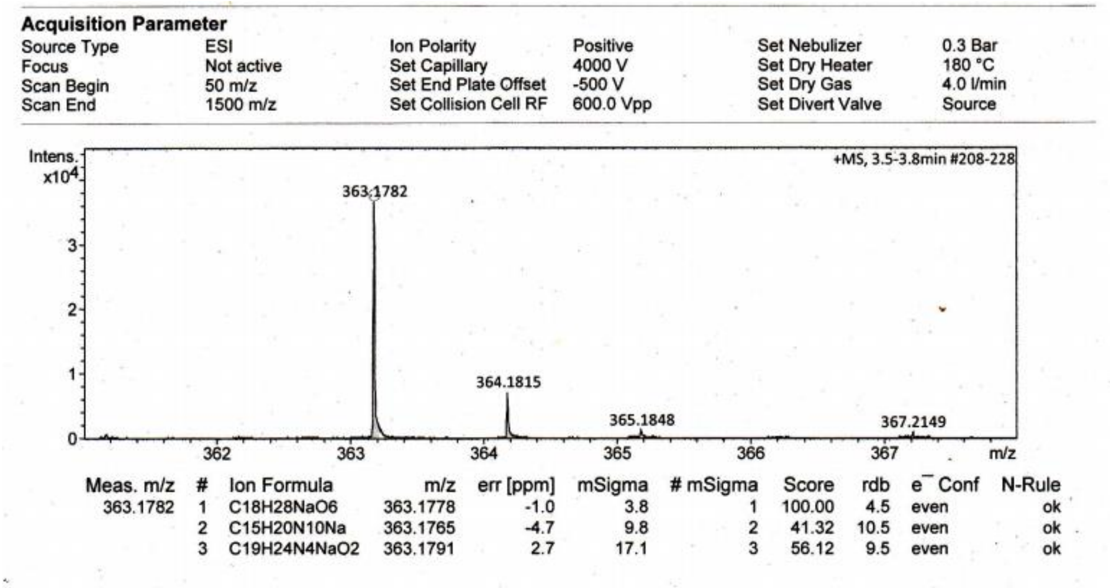

Figure S9. The HRESIMS of compound **2**.

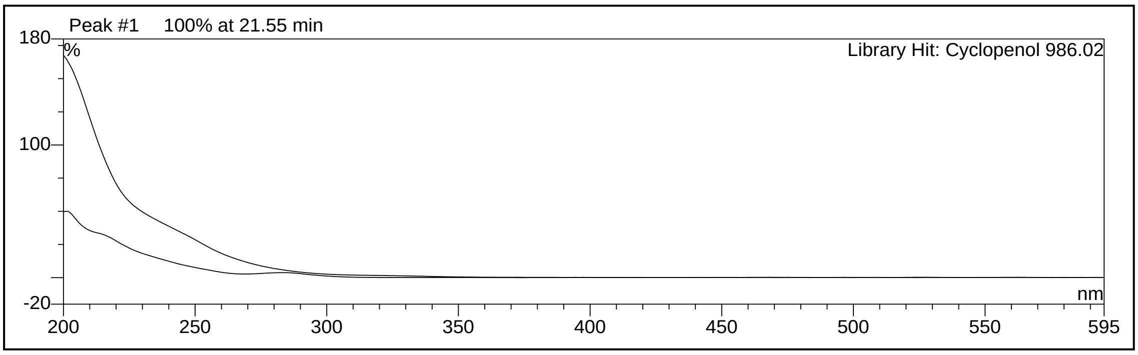

Figure S10. The UV spectrum of compound **2**.

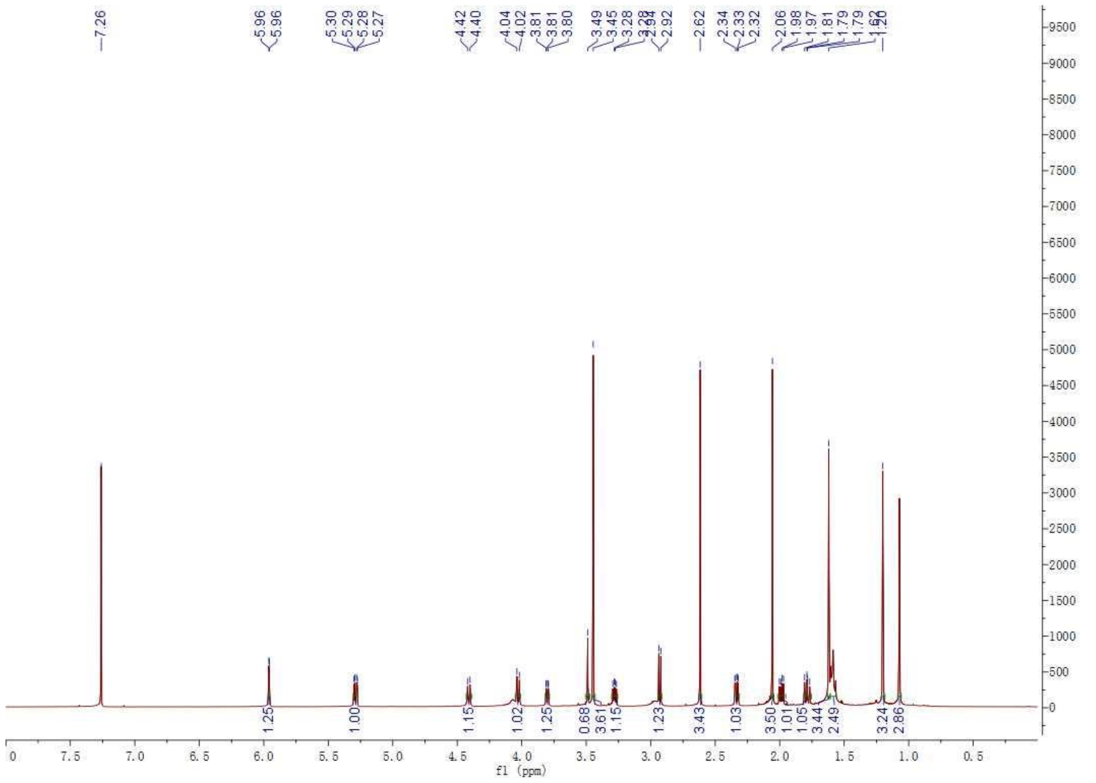

Figure S11. The ^{1}H-NMR (600 MHz, CDCl3) spectrum of compound **2**.

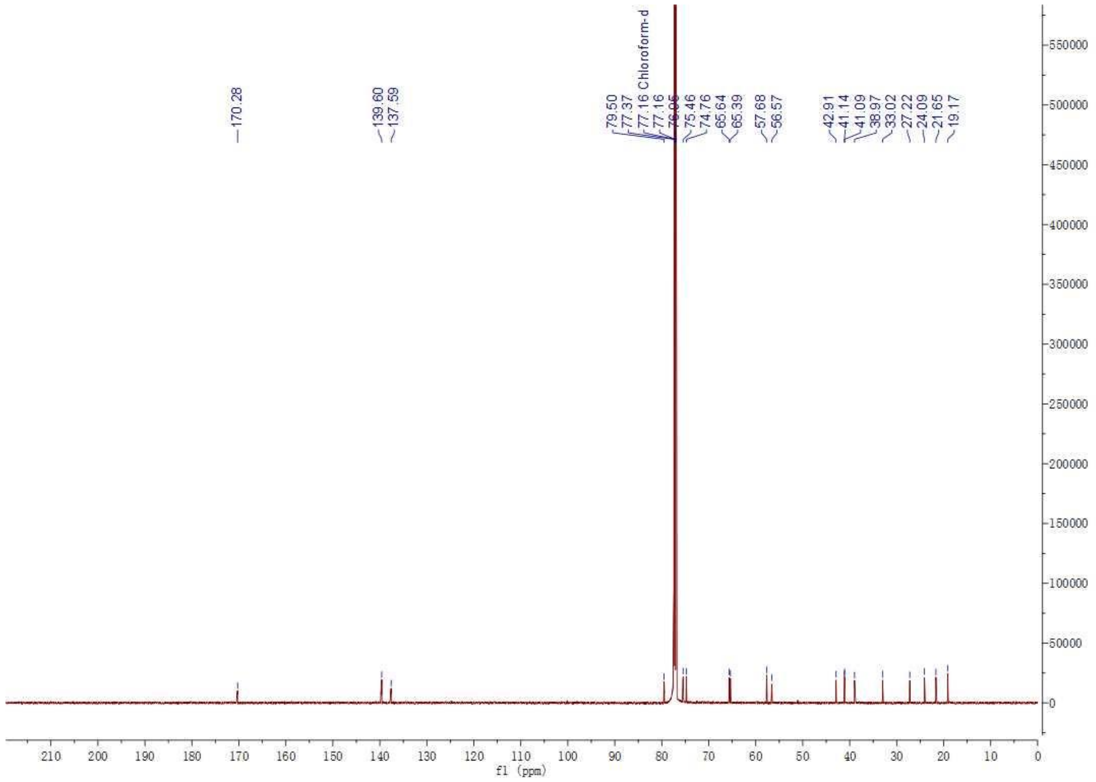

Figure S12. The ^{13}C-NMR (150 MHz, CDCl3) spectrum of compound **2**.

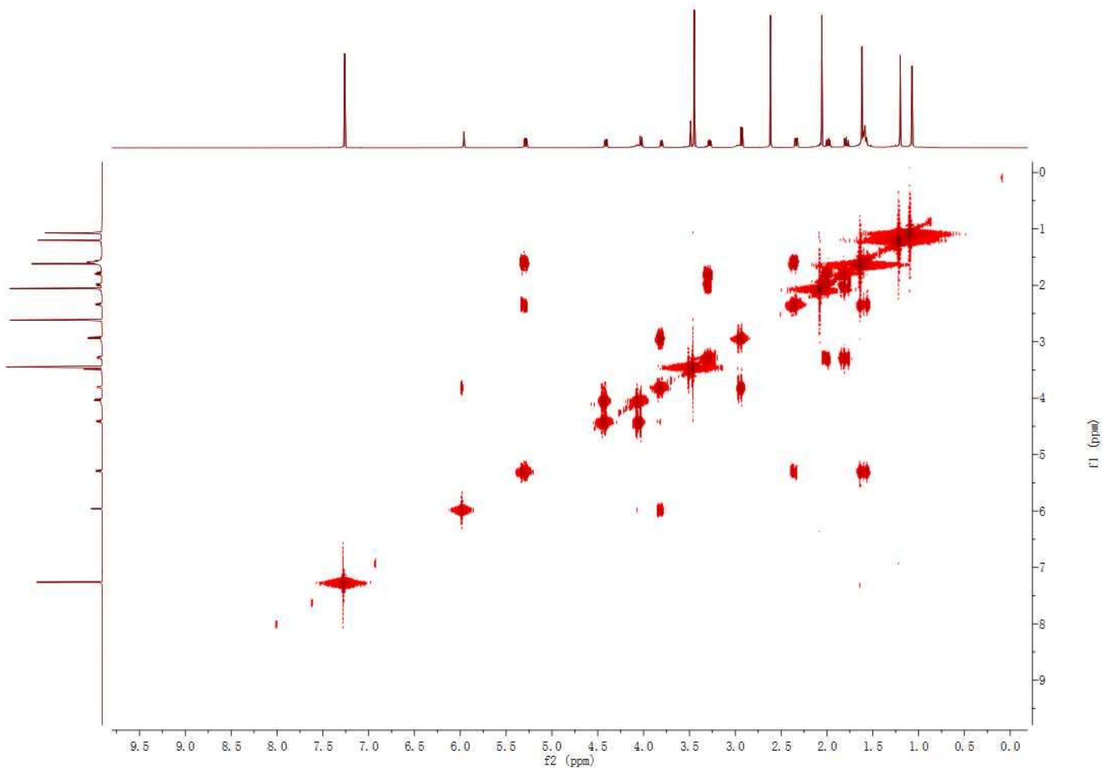

Figure S13. The ^{1}H-H COSY (600 MHz, $CDCl_3$) spectrum of compound **2**.

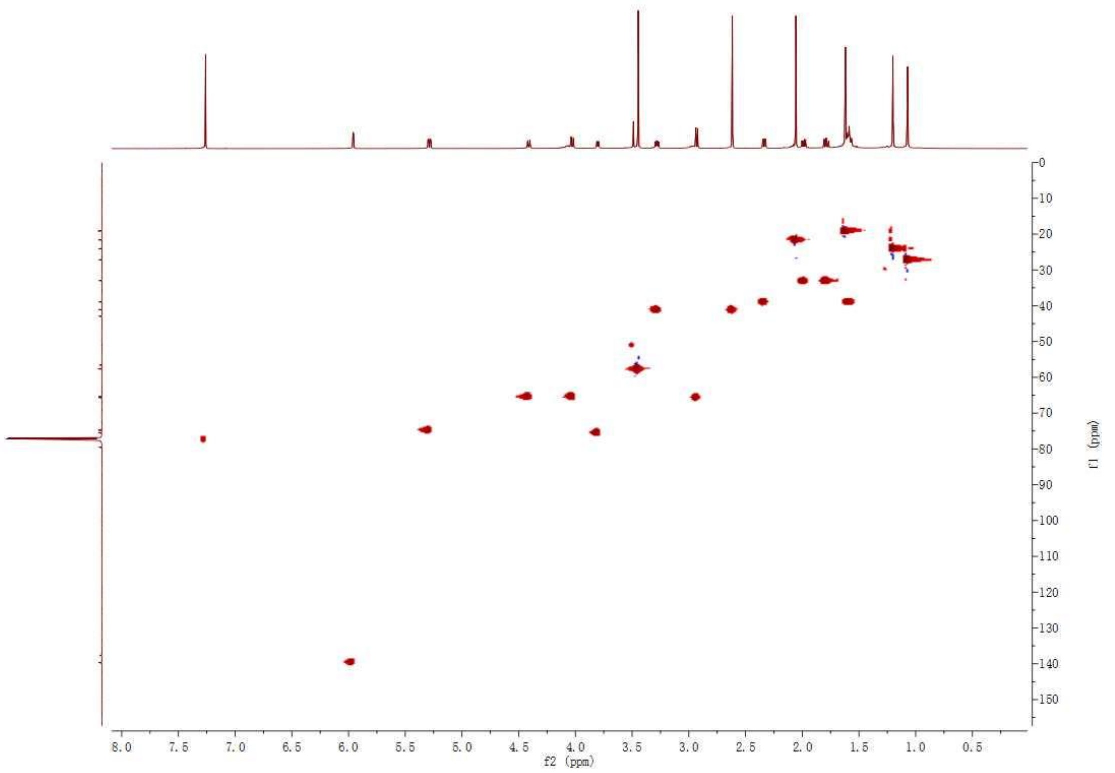

Figure S14. The HSQC (600 MHz, $CDCl_3$) spectrum of compound **2**.

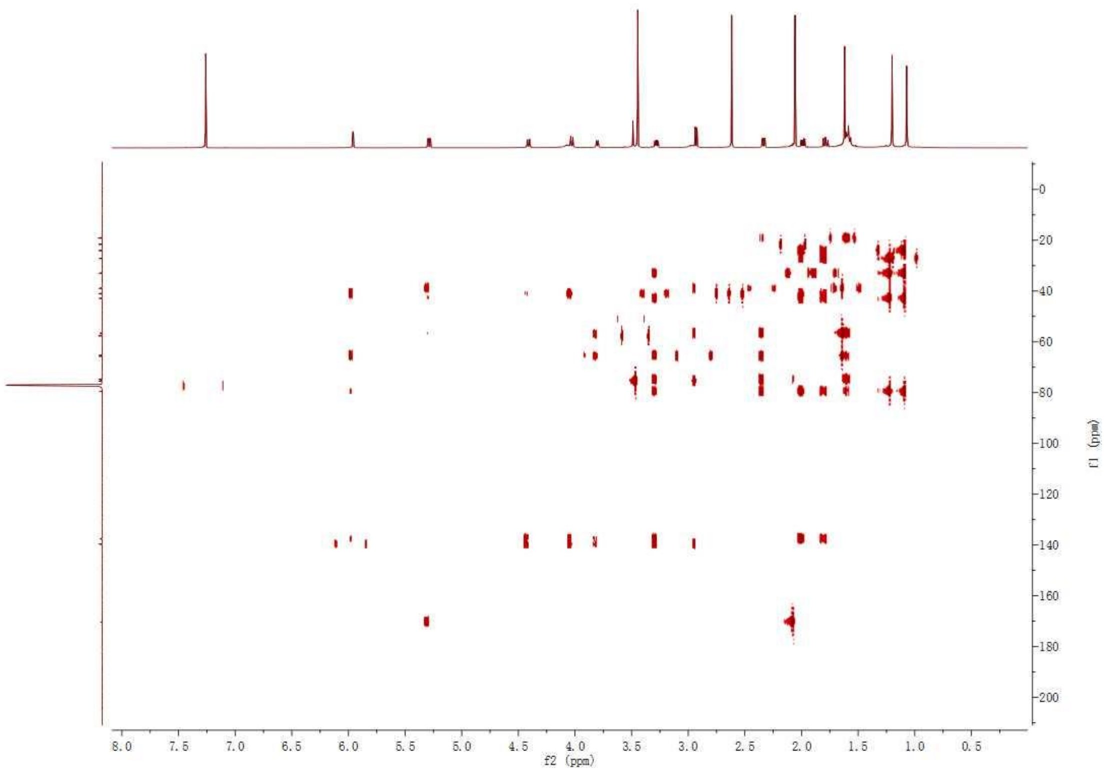

Figure S15. The HMBC (600 MHz, $CDCl_3$) spectrum of compound **2**.

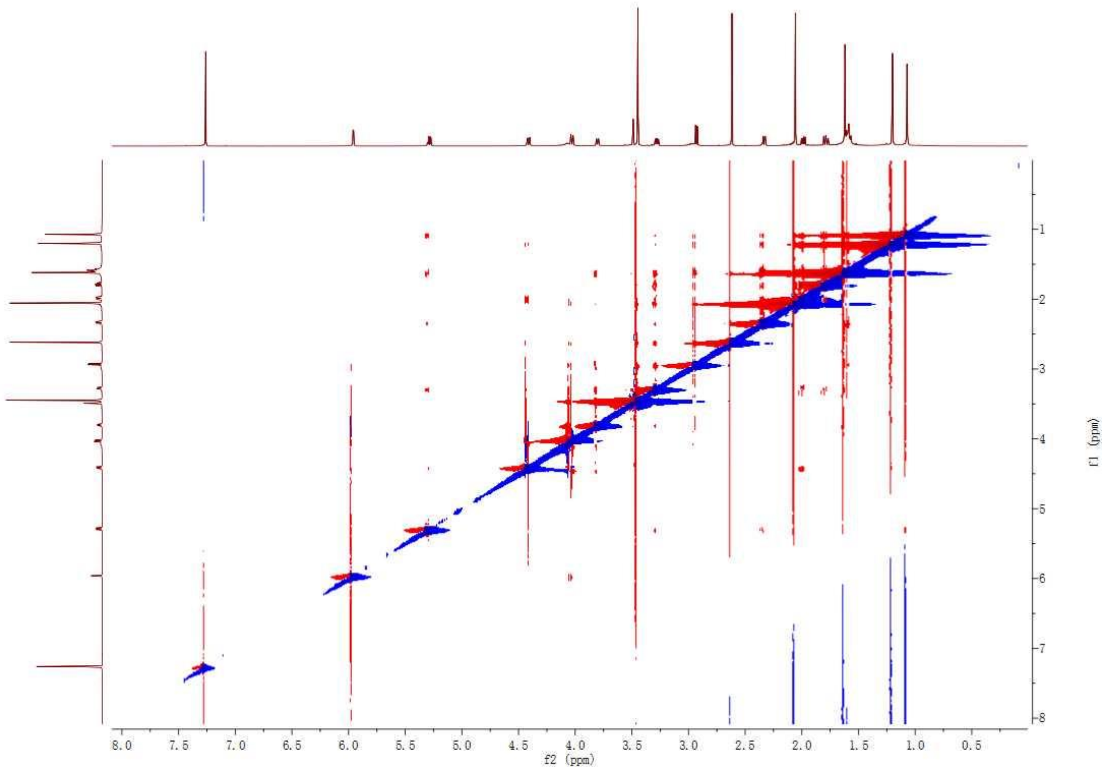

Figure S16. The ROESY (600 MHz, $CDCl_3$) spectrum of compound **2**.

Acquisition Parameter

Source Type	ESI	Ion Polarity	Positive	Set Nebulizer	0.3 Bar
Focus	Not active	Set Capillary	4000 V	Set Dry Heater	180 °C
Scan Begin	50 m/z	Set End Plate Offset	-500 V	Set Dry Gas	4.0 l/min
Scan End	1500 m/z	Set Collision Cell RF	600.0 Vpp	Set Divert Valve	Source

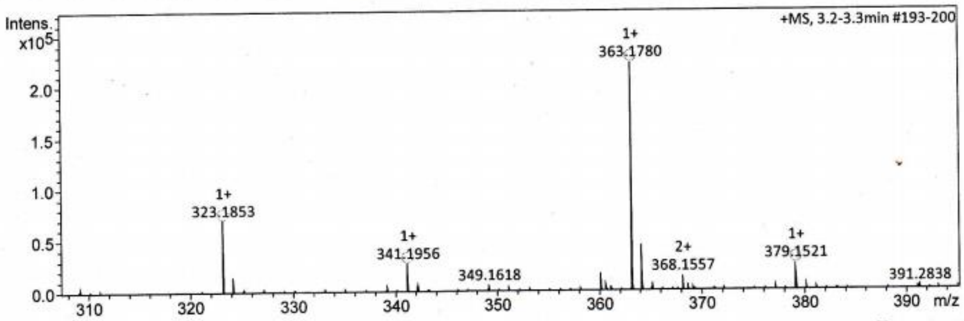

Meas. m/z	#	Ion Formula	m/z	err [ppm]	mSigma	# mSigma	Score	rdb	e⁻ Conf	N-Rule
323.1853	1	C18H27O5	323.1853	0.1	5.3	1	100.00	5.5	even	ok
341.1956	1	C18H29O6	341.1959	0.7	1.5	1	100.00	4.5	even	ok
363.1780	1	C16H23N6O4	363.1775	-1.2	2.2	1	88.40	8.5	even	ok
	2	C18H28NaO6	363.1778	-0.4	2.4	2	100.00	4.5	even	ok
379.1521	1	C21H24NaO5	379.1516	-1.2	61.9	1	100.00	9.5	even	ok
	2	C19H19N6O3	379.1513	-2.0	64.1	2	80.52	13.5	even	ok
703.3666	1	C34H51N6O10	703.3661	-0.7	9.3	1	89.02	12.5	even	ok
	2	C36H56NaO12	703.3664	-0.3	10.0	2	100.00	8.5	even	ok
	3	C35H47N10O6	703.3675	1.2	21.0	3	58.62	17.5	even	ok
	4	C37H52N4NaO8	703.3677	1.6	21.6	4	49.34	13.5	even	ok
	5	C34H44N14NaO2	703.3664	-0.3	22.5	5	77.22	19.5	even	ok
	6	C32H39N20	703.3661	-0.7	22.6	6	67.78	23.5	even	ok
	7	C49H48N2NaO	703.3659	-1.0	84.6	7	8.42	26.5	even	ok

Figure S17. The HRESIMS of compound **3**.

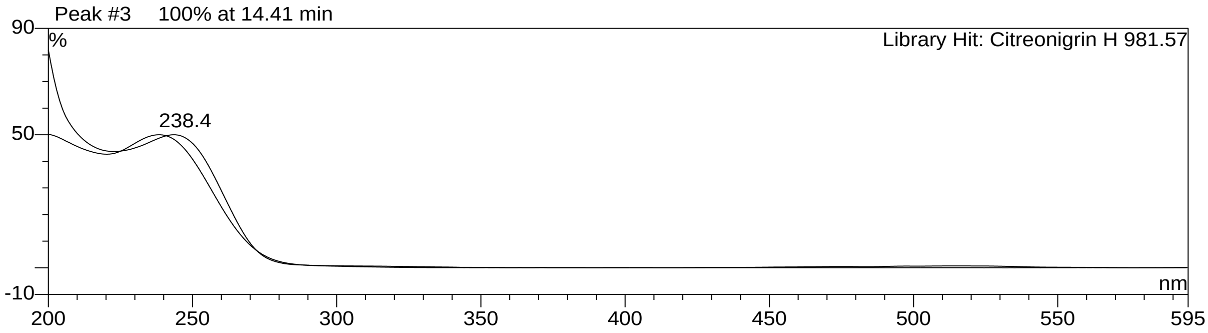

Figure S18. The UV spectrum of compound **3**.

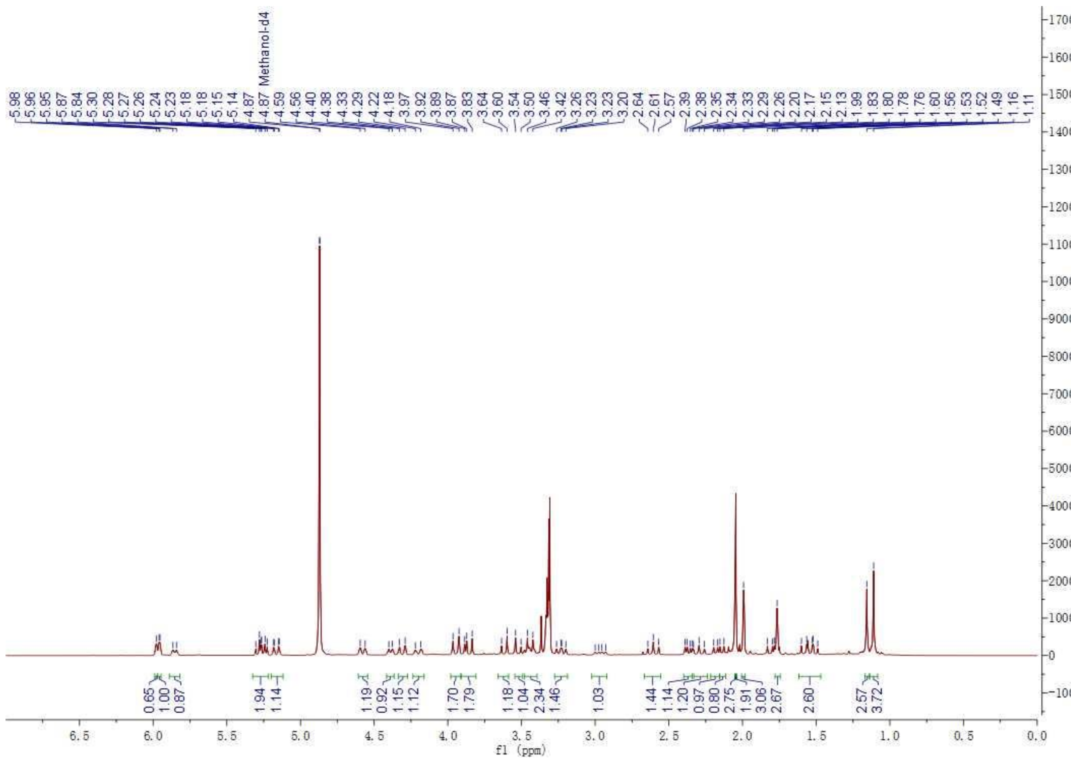

Figure S19. The ^{1}H-NMR (600 MHz, CD_3OD) spectrum of compound **3**.

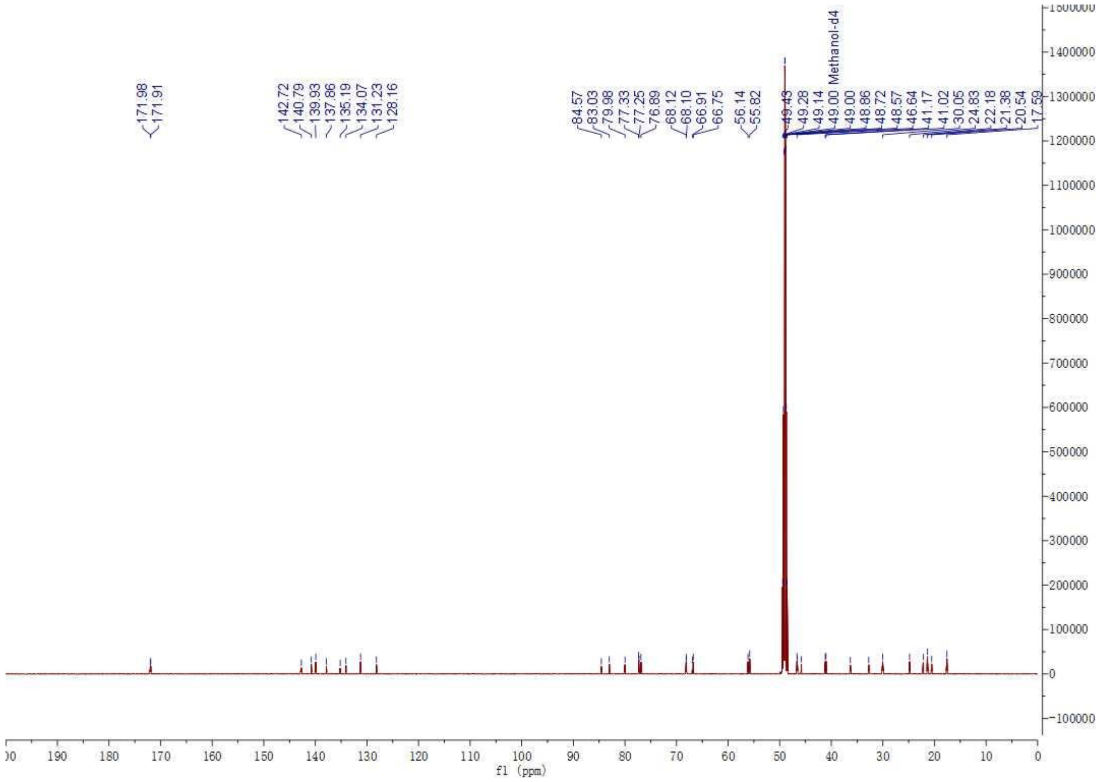

Figure S20. The ^{13}C-NMR (150 MHz, CD_3OD) spectrum of compound **3**.

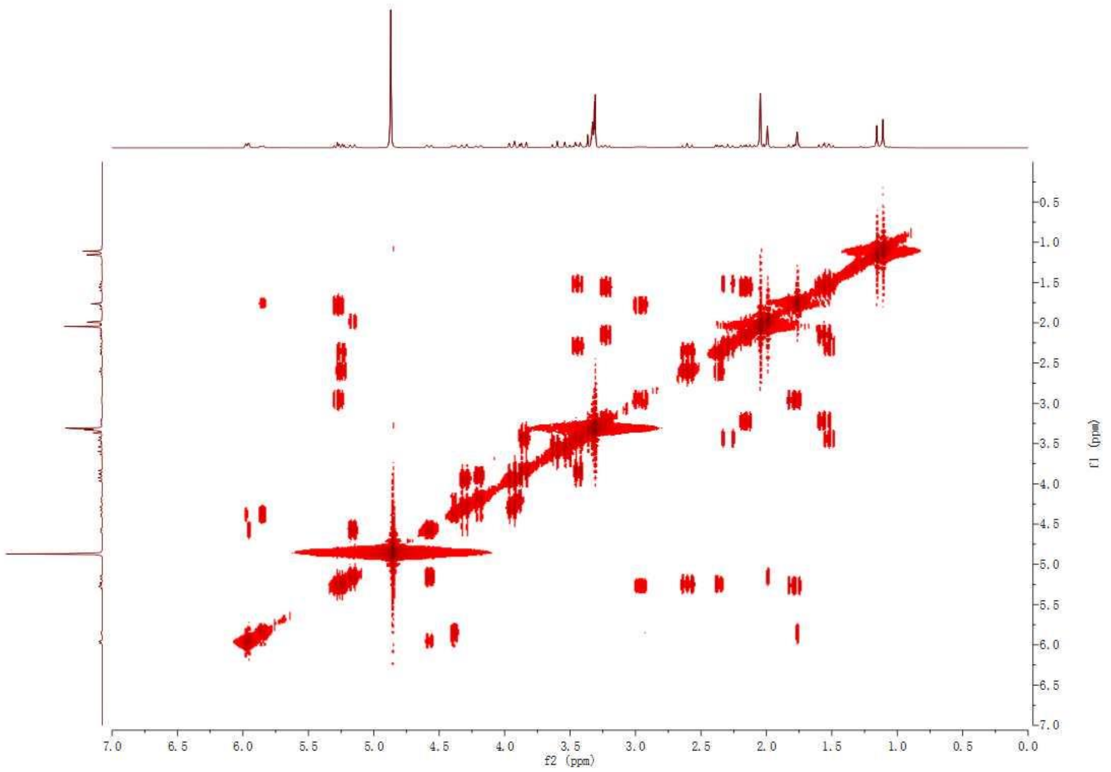

Figure S21. The ^{1}H-^{1}H COSY (600 MHz, CD_3OD) spectrum of compound **3**.

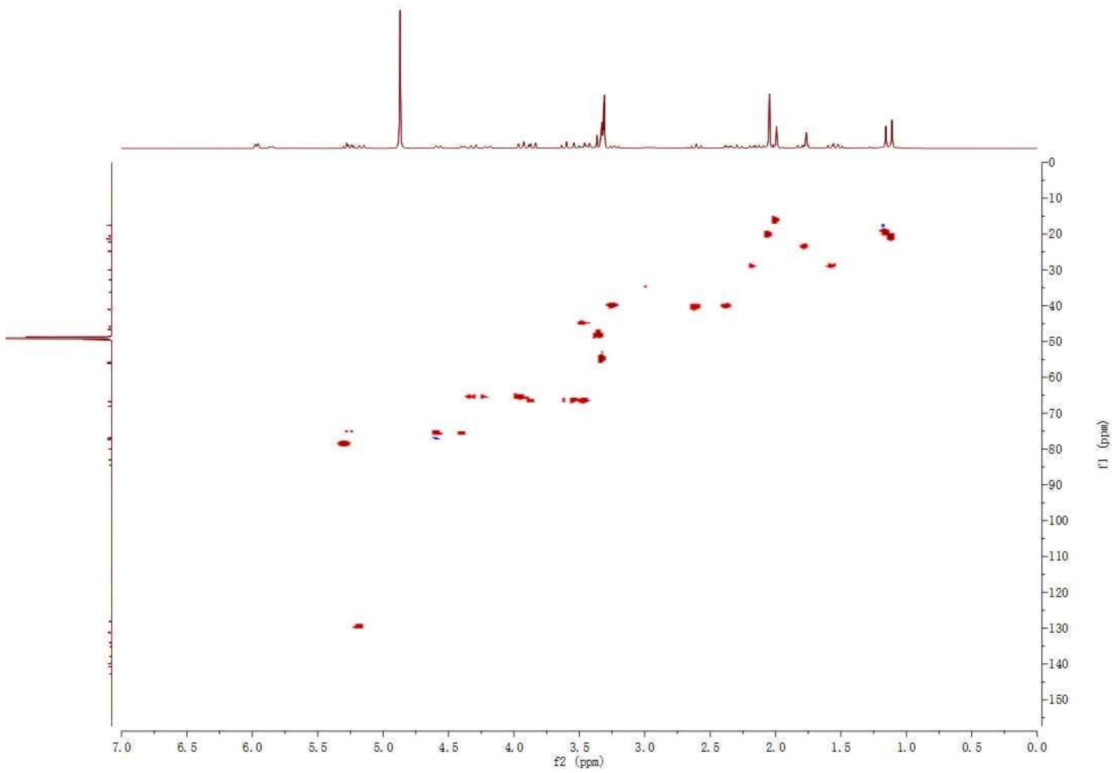

Figure S22. The HSQC (600 MHz, CD_3OD) spectrum of compound **3**.

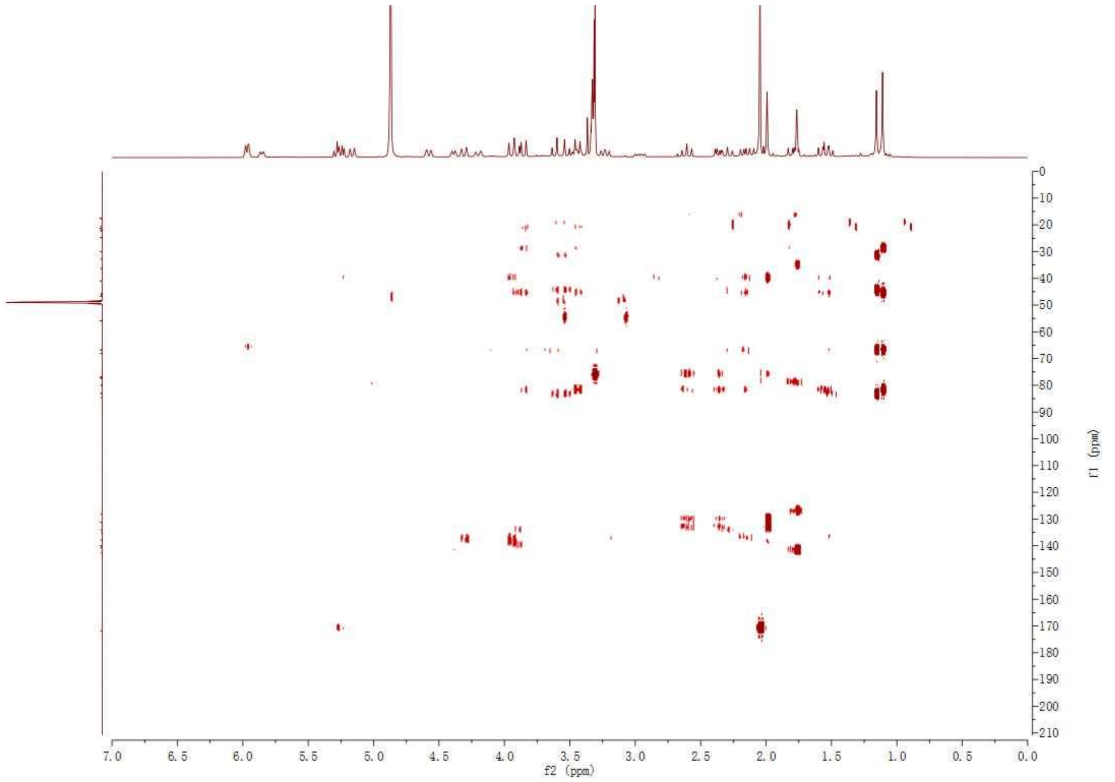

Figure S23. The HMBC (600 MHz, CD_3OD) spectrum of compound **3**.

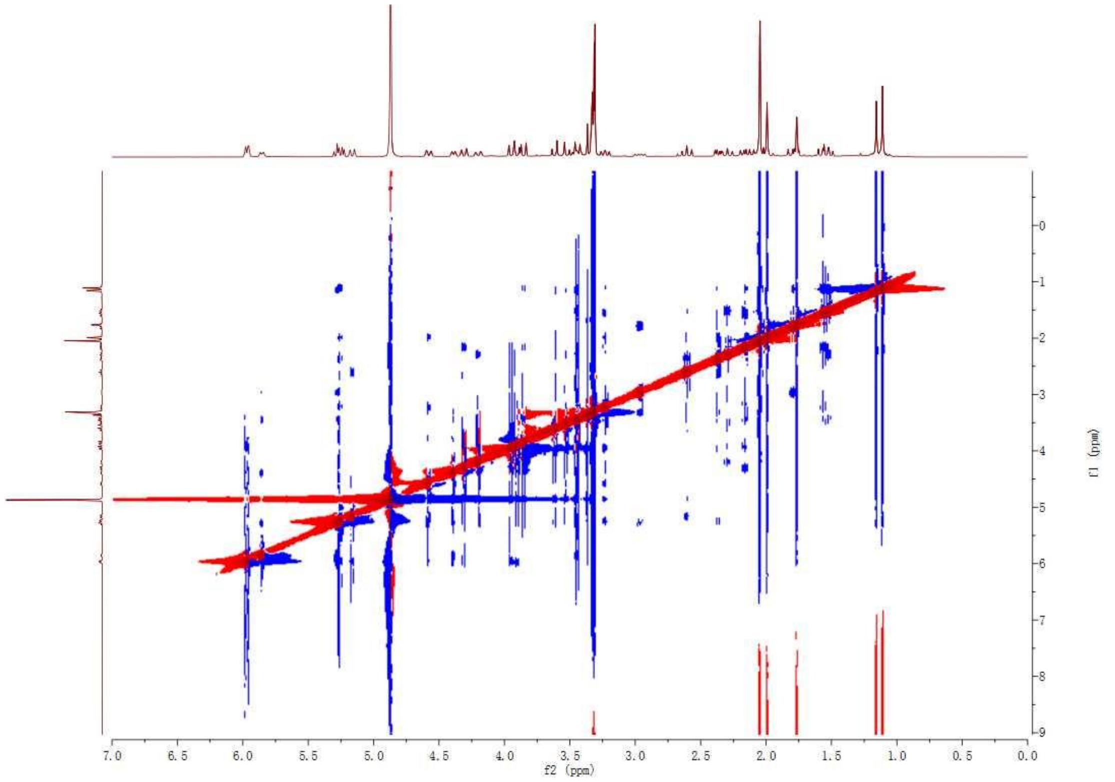

Figure S24. The ROESY (600 MHz, CD_3OD) spectrum of compound **3**.

Acquisition Parameter

Source Type	ESI	Ion Polarity	Positive	Set Nebulizer	0.3 Bar
Focus	Not active	Set Capillary	4000 V	Set Dry Heater	180 °C
Scan Begin	50 m/z	Set End Plate Offset	-500 V	Set Dry Gas	4.0 l/min
Scan End	1500 m/z	Set Collision Cell RF	600.0 Vpp	Set Divert Valve	Source

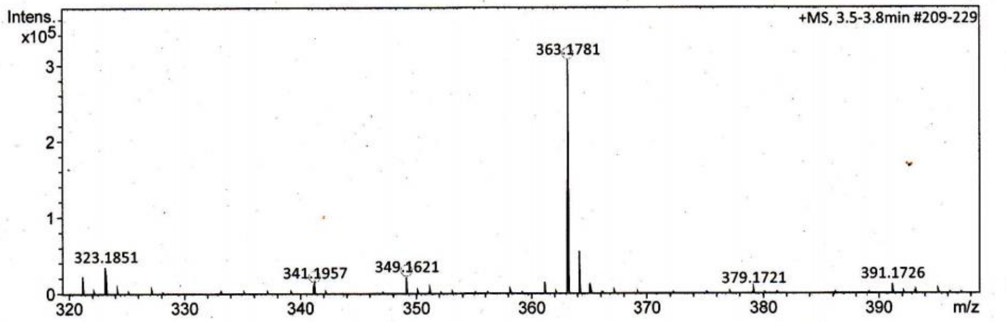

Meas. m/z	#	Ion Formula	m/z	err [ppm]	mSigma	# mSigma	Score	rdb	e⁻ Conf	N-Rule
323.1851	1	C18H27O5	323.1853	0.7	8.2	1	100.00	5.5	even	ok
341.1957	1	C18H29O6	341.1959	0.5	4.5	1	100.00	4.5	even	ok
349.1621	1	C17H26NaO6	349.1622	0.2	152.9	1	100.00	4.5	even	ok
363.1781	1	C18H28NaO6	363.1778	-0.9	11.6	1	100.00	4.5	even	ok

Figure S25. The HRESIMS of compound **4**.

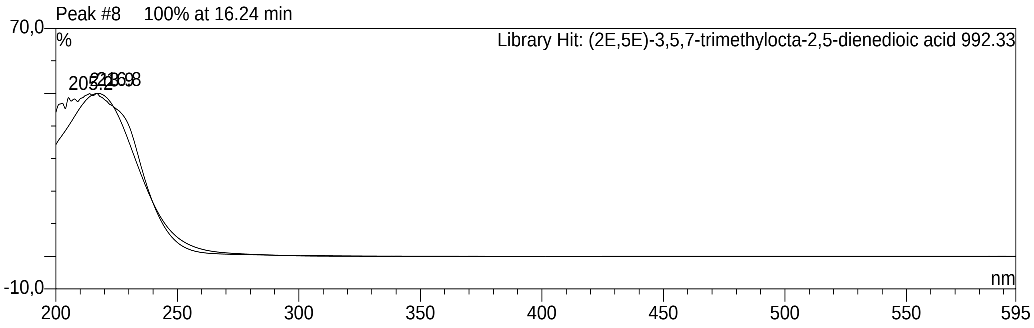

Figure S26. The UV spectrum of compound **4**.

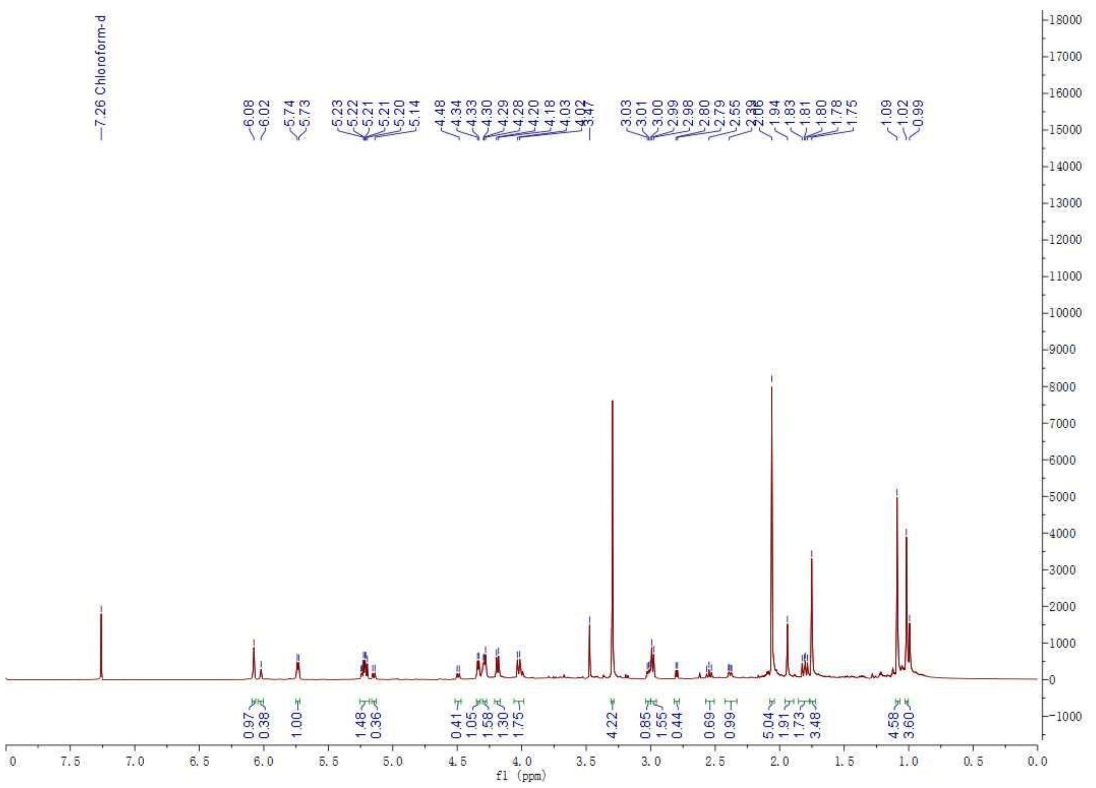

Figure S27. The ^{1}H-NMR (600 MHz, $CDCl_3$) spectrum of compound **4**.

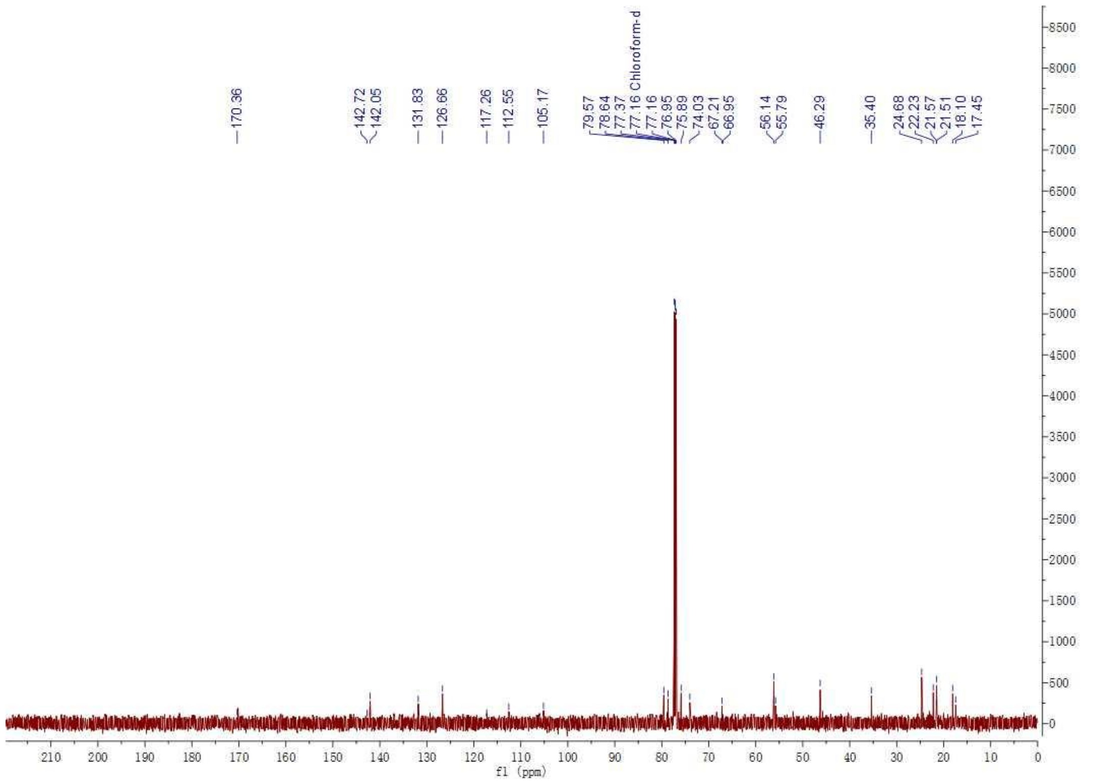

Figure S28. The ^{13}C-NMR (150 MHz, $CDCl_3$) spectrum of compound **4**.

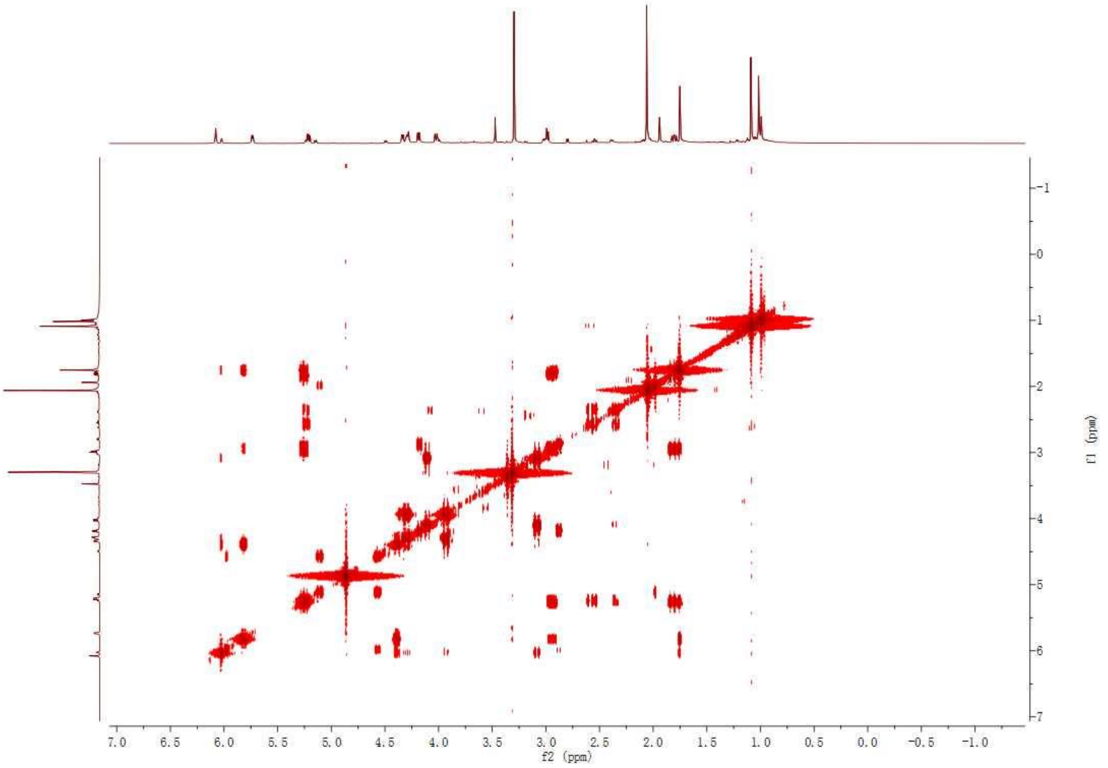

Figure S29. The ^{1}H-^{1}H COSY (600 MHz, $CDCl_3$) spectrum of compound **4**.

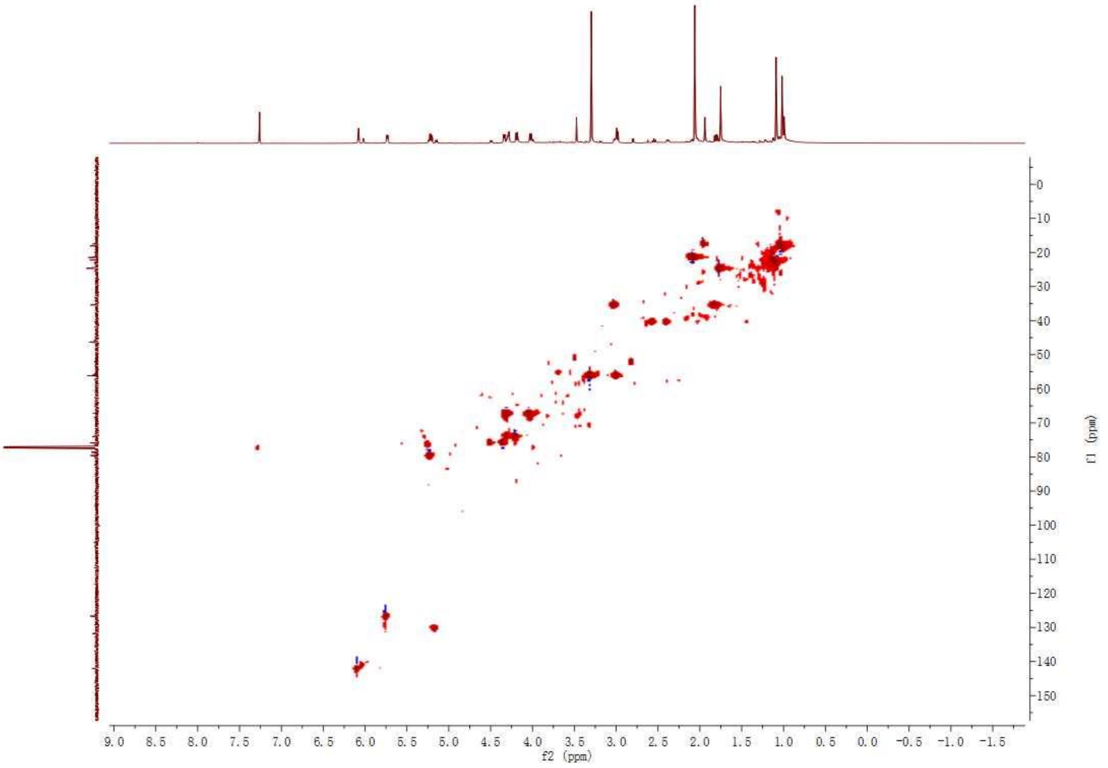

Figure S30. The HSQC (150 MHz, $CDCl_3$) spectrum of compound **4**.

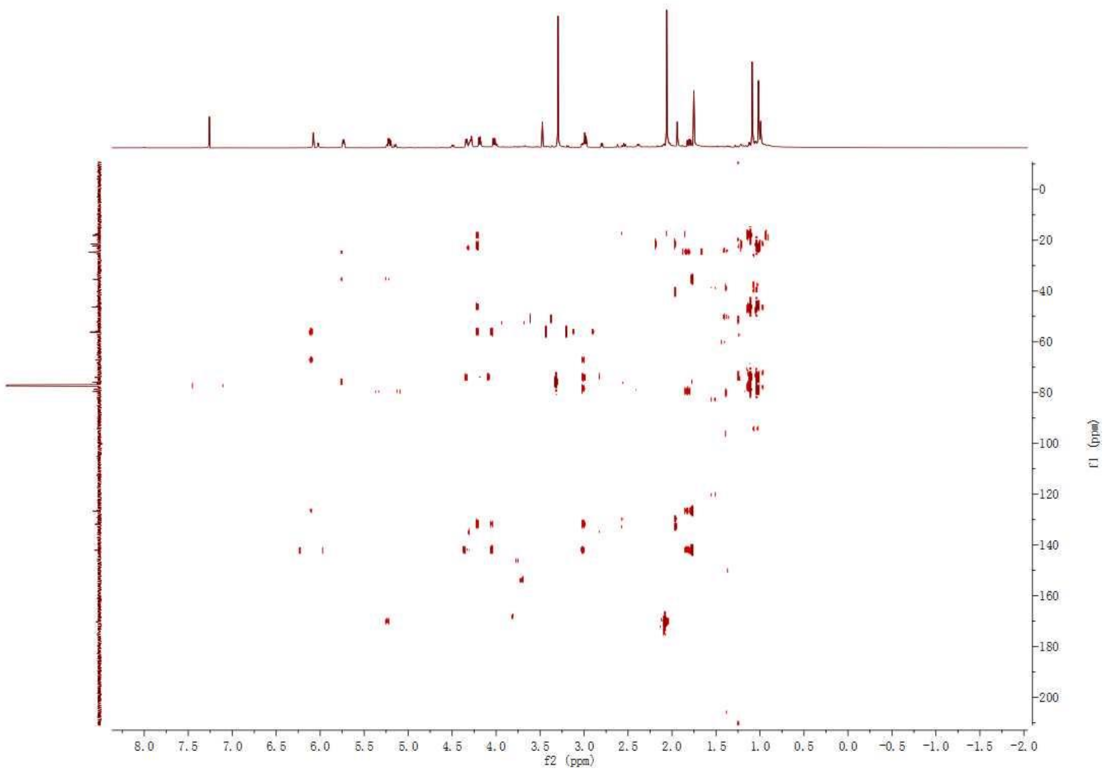

Figure S31. The HMBC (600MHz, $CDCl_3$) spectrum of compound **4**.

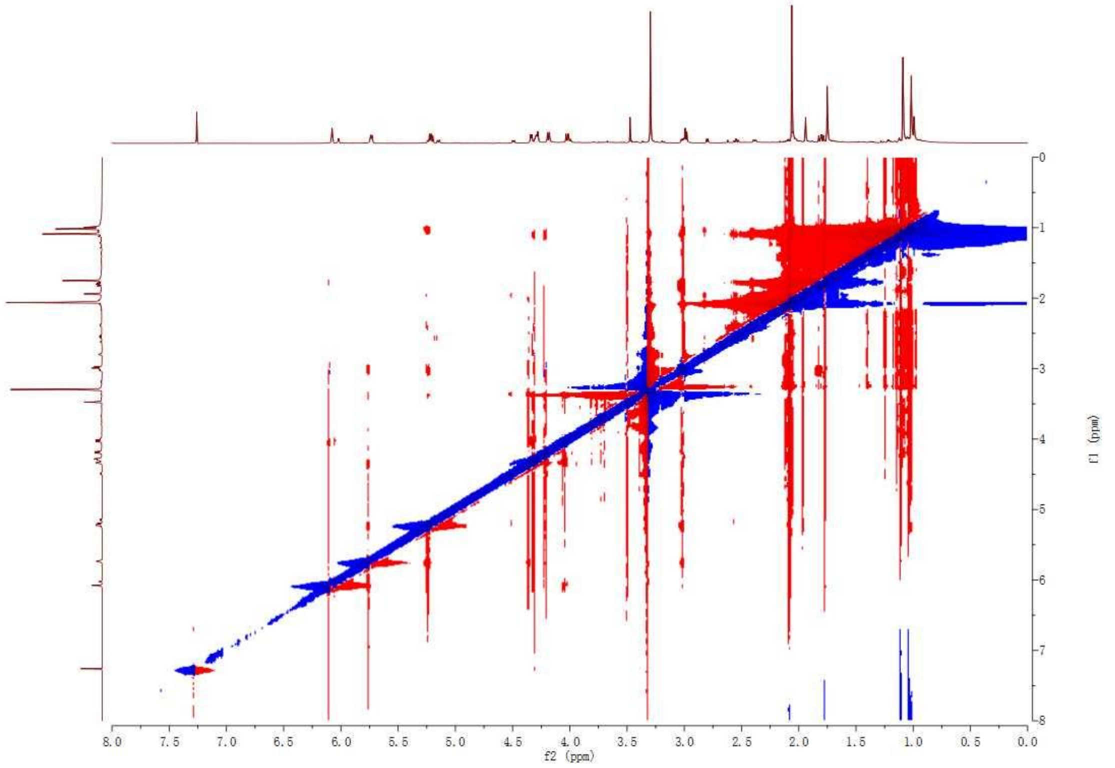

Figure S32. The ROESY (600 MHz, $CDCl_3$) spectrum of compound **4**.

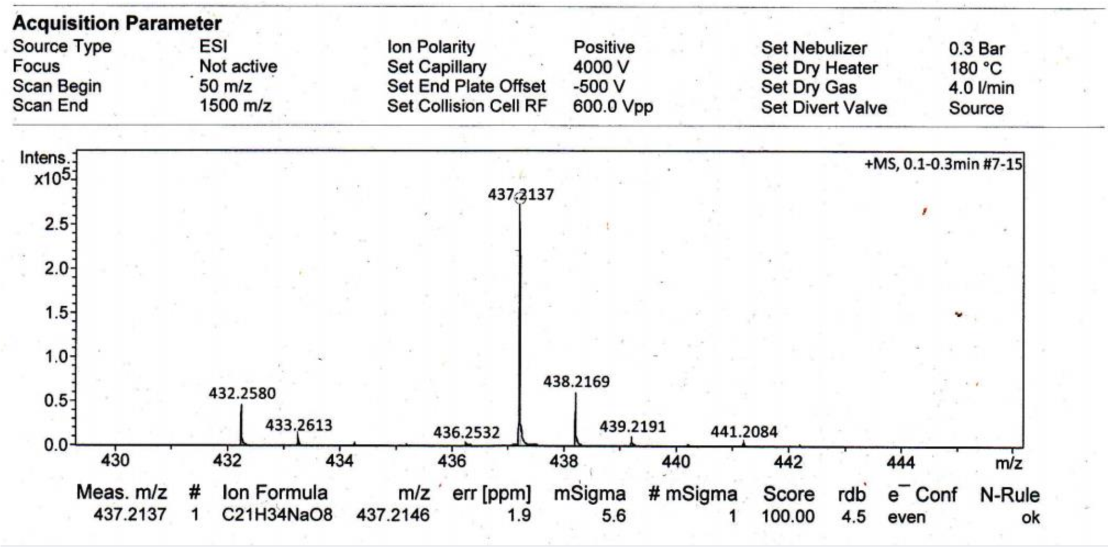

Figure S33. The HRESIMS of compound **5**.

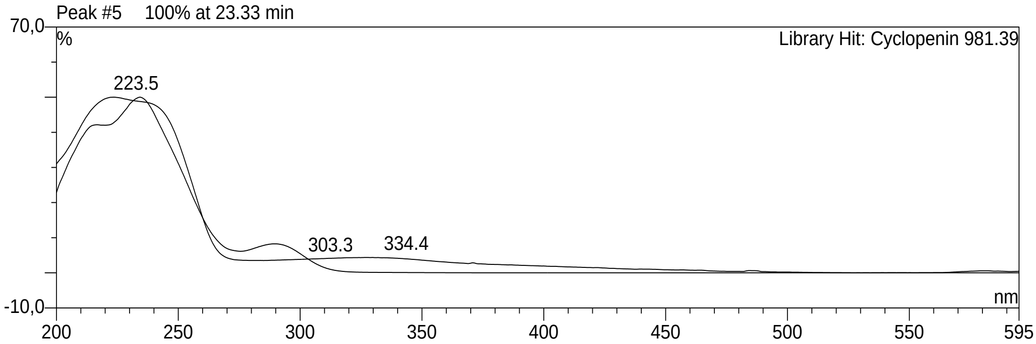

Figure S34. The UV spectrum of compound **5**.

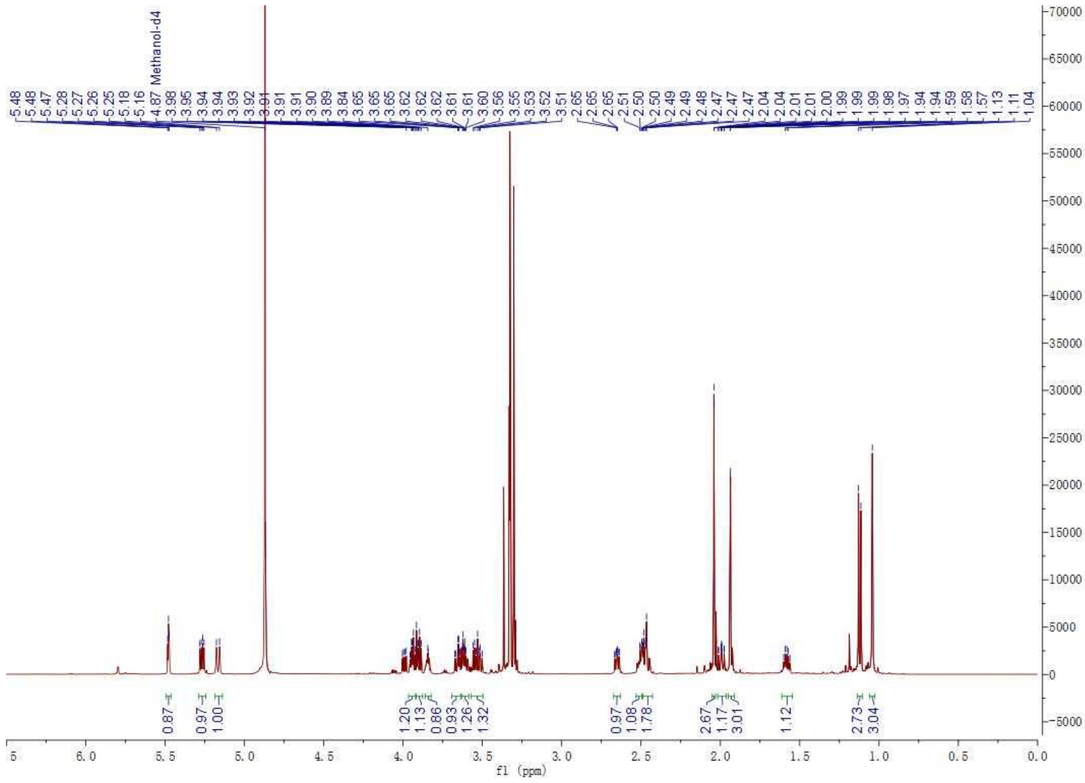

Figure S35. The ^{1}H-NMR (600 MHz, CD_3OD) spectrum of compound **5**.

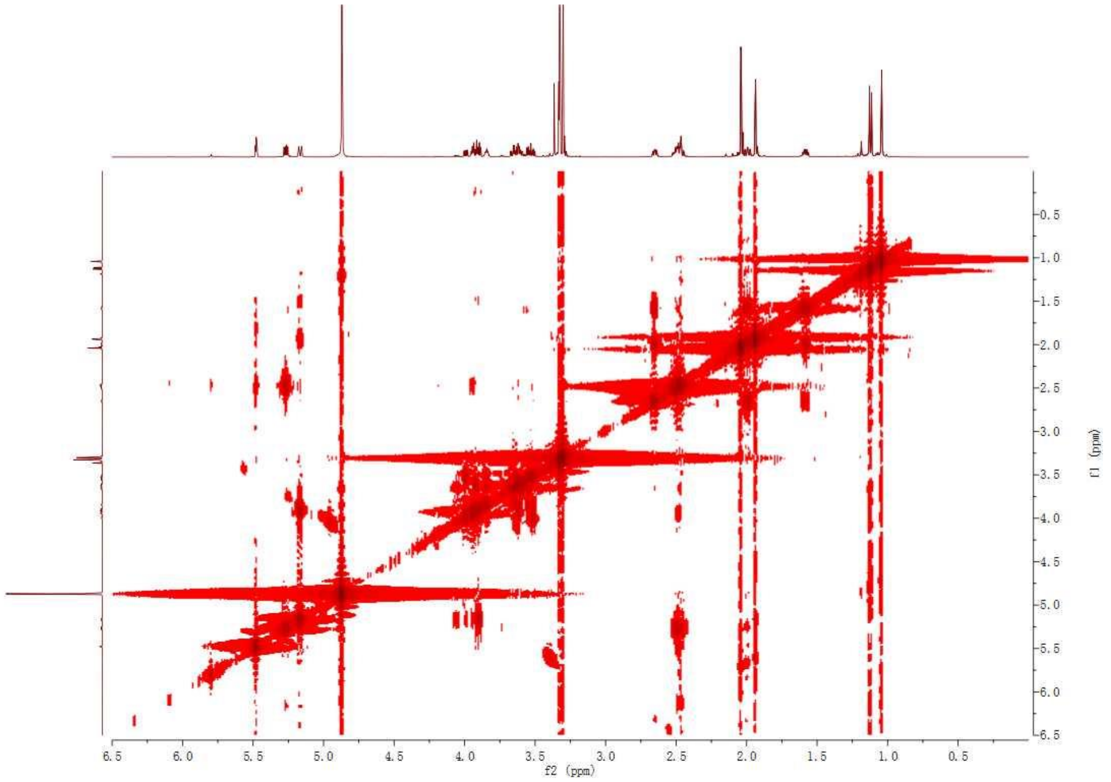

Figure S36. The ^{1}H-^{1}H COSY (600 MHz, CD_3OD) spectrum of compound **5**.

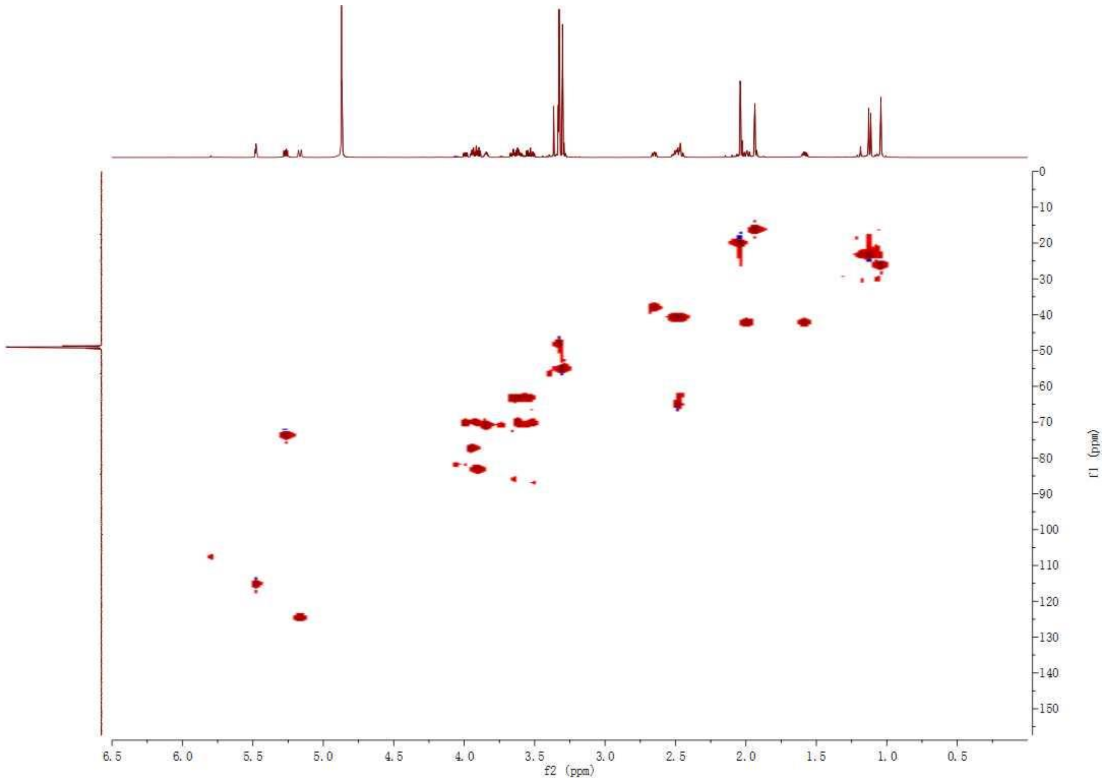

Figure S37. The HSQC (600 MHz, CD_3OD) spectrum of compound **5**.

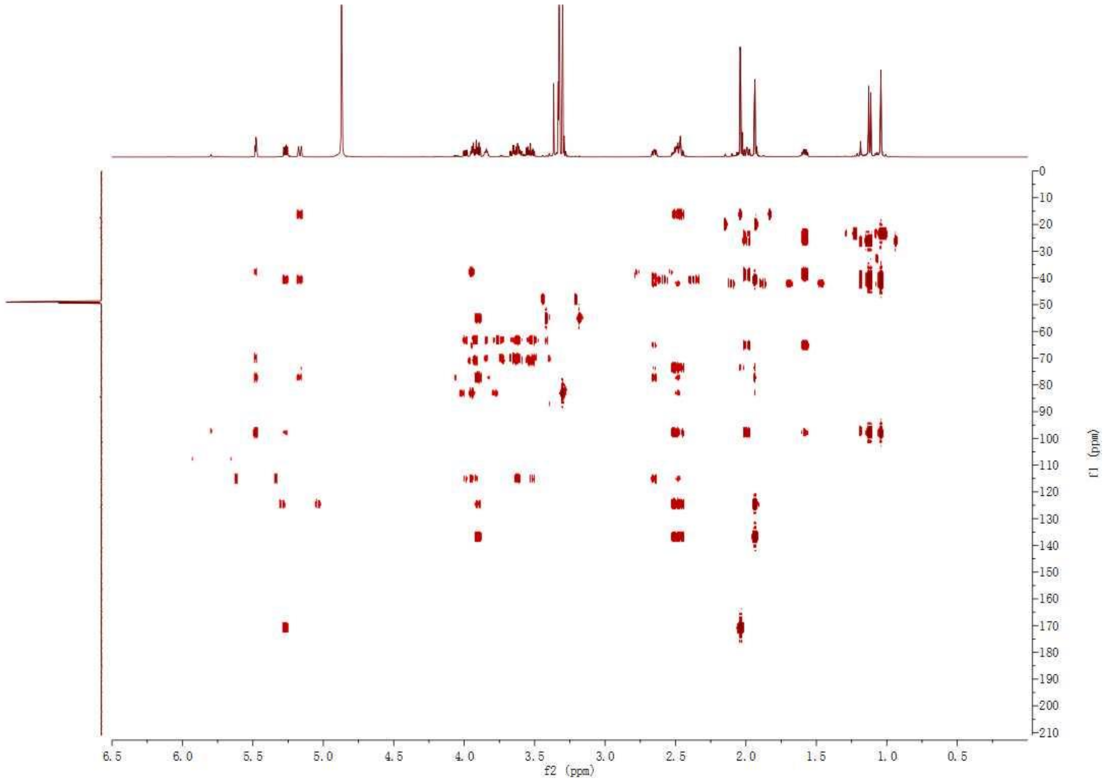

Figure S38. The HMBC (600MHz, CD_3OD) spectrum of compound **5**.

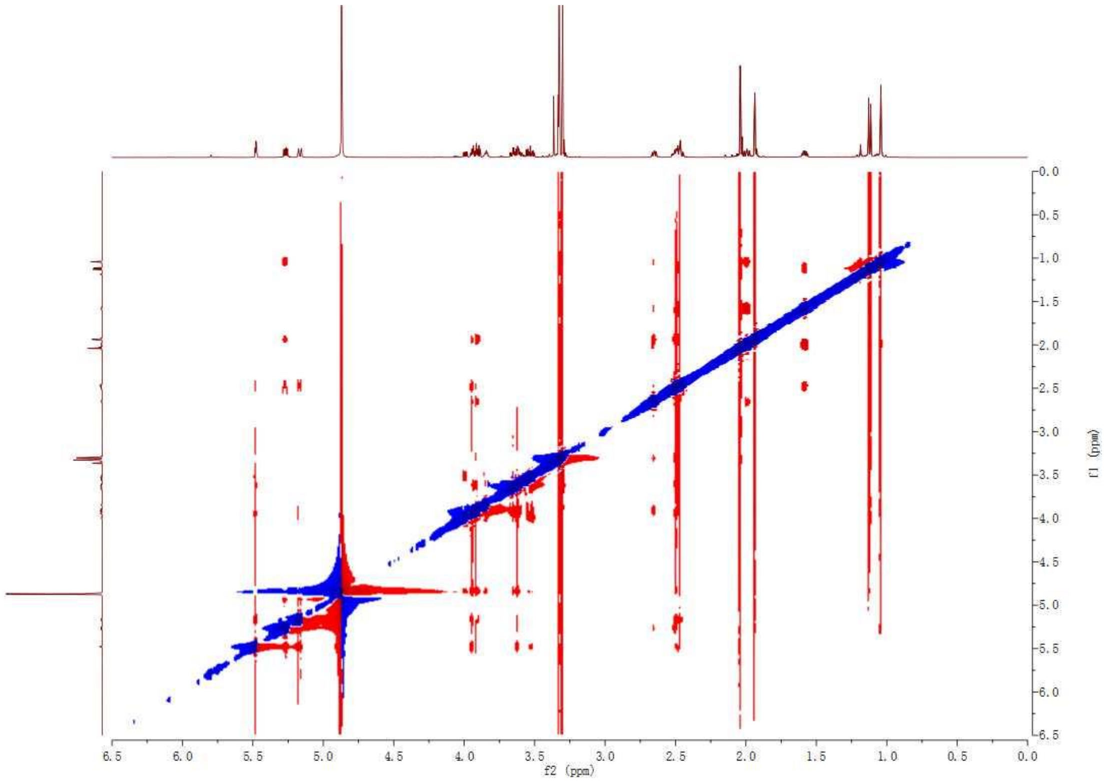

Figure S39. The ROESY (600 MHz, CD_3OD) spectrum of compound **5**.

Acquisition Parameter

Source Type	ESI	Ion Polarity	Positive	Set Nebulizer	0.3 Bar
Focus	Not active	Set Capillary	4000 V	Set Dry Heater	180 °C
Scan Begin	50 m/z	Set End Plate Offset	-500 V	Set Dry Gas	4.0 l/min
Scan End	1500 m/z	Set Collision Cell RF	600.0 Vpp	Set Divert Valve	Source

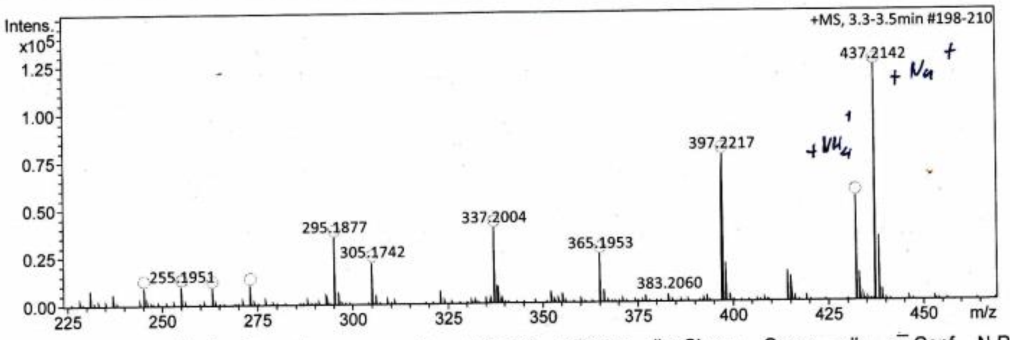

Meas. m/z	#	Ion Formula	m/z	err [ppm]	mSigma	# mSigma	Score	rdb	e⁻ Conf	N-Rule
245.1533	1	C16H21O2	245.1536	1.3	58.4	1	100.00	6.5	even	ok
255.1951	1	C15H27O3	255.1955	1.5	11.0	1	100.00	2.5	even	ok
263.1637	1	C16H23O3	263.1642	1.7	14.8	1	100.00	5.5	even	ok
273.2058	1	C15H29O4	273.2060	0.9	9.3	1	100.00	1.5	even	ok
295.1877	1	C15H28NaO4	295.1880	0.9	14.1	1	92.85	1.5	even	ok
	2	C13H23N6O2	295.1877	-0.1	15.2	2	100.00	5.5	even	ok
305.1742	1	C18H25O4	305.1747	1.7	10.9	1	100.00	6.5	even	ok
	2	C17H22N4Na	305.1737	-1.8	11.8	2	96.14	8.5	even	ok
337.2004	1	C19H29O5	337.2010	1.5	14.2	1	100.00	5.5	even	ok
	2	C18H26N4NaO	337.1999	-1.7	16.2	2	93.43	7.5	even	ok
365.1953	1	C20H29O6	365.1959	1.6	19.1	1	97.97	6.5	even	ok
	2	C19H26N4NaO2	365.1948	-1.4	19.7	2	100.00	8.5	even	ok
397.2217	1	C21H33O7	397.2221	0.9	17.4	1	100.00	5.5	even	ok
	2	C20H30N4NaO3	397.2210	-1.8	18.0	2	83.02	7.5	even	ok
432.2587	1	C21H38NO8	432.2592	1.2	16.2	1	100.00	3.5	even	ok
	2	C18H30N11O2	432.2578	-1.9	19.1	2	81.21	9.5	even	ok
437.2142	1	C21H34NaO8	437.2146	0.9	22.4	1	100.00	4.5	even	ok

Figure S40. The HRESIMS of compound **6**.

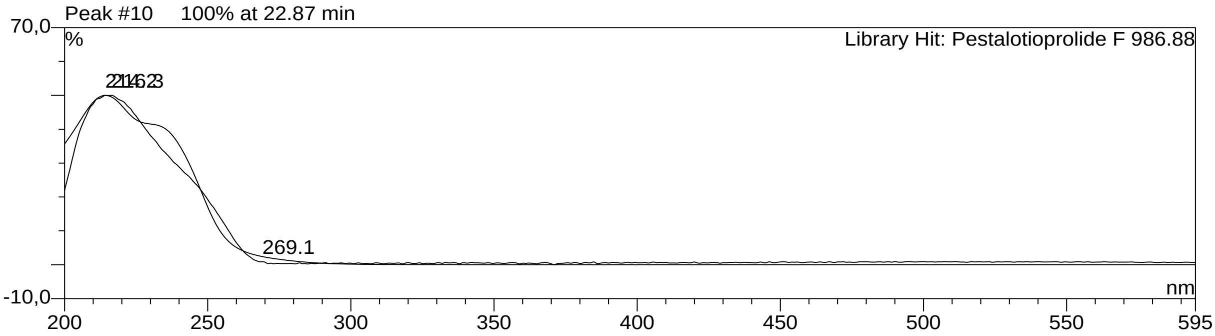

Figure S41. The UV spectrum of compound **6**.

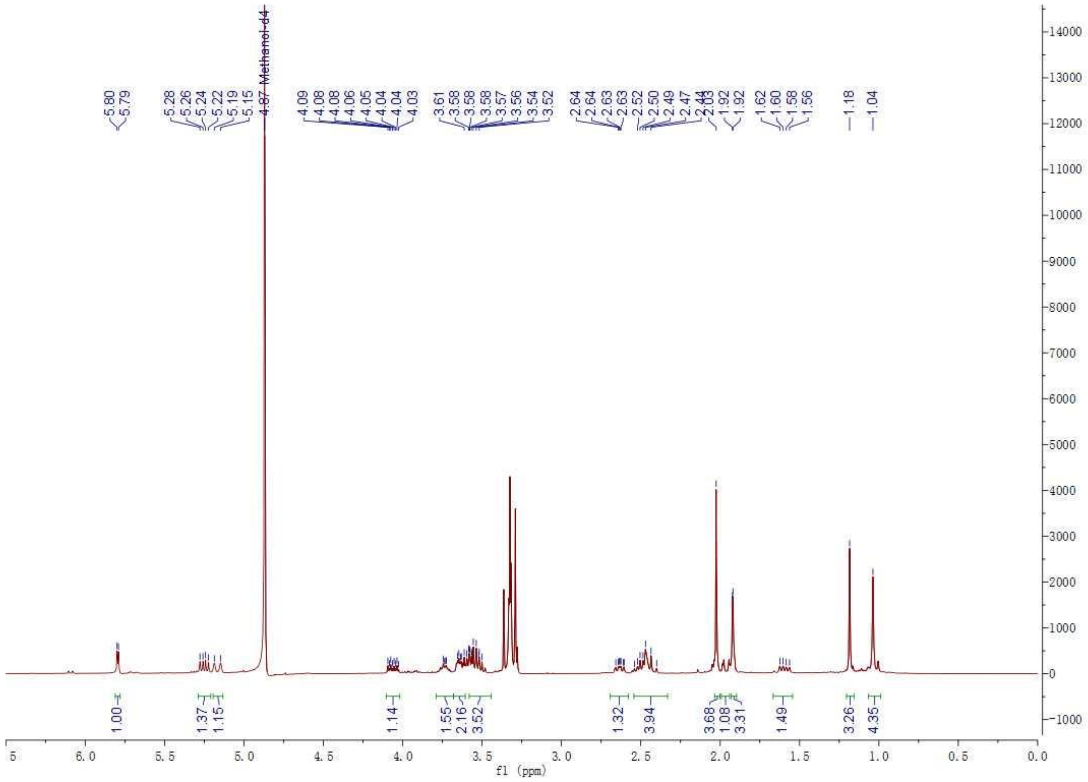

Figure S42. The ^{1}H-NMR (600 MHz, CD_3OD) spectrum of compound **6**.

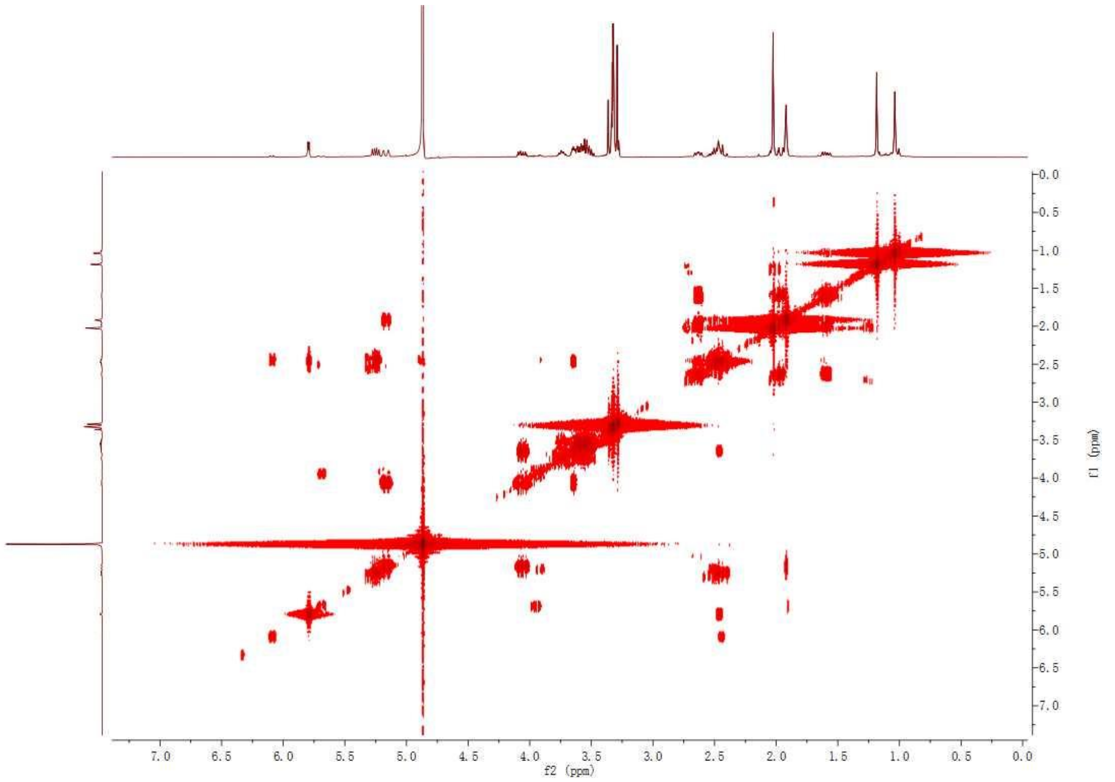

Figure S43. The ^{1}H-^{1}H COSY (600 MHz, CD_3OD) spectrum of compound **6**.

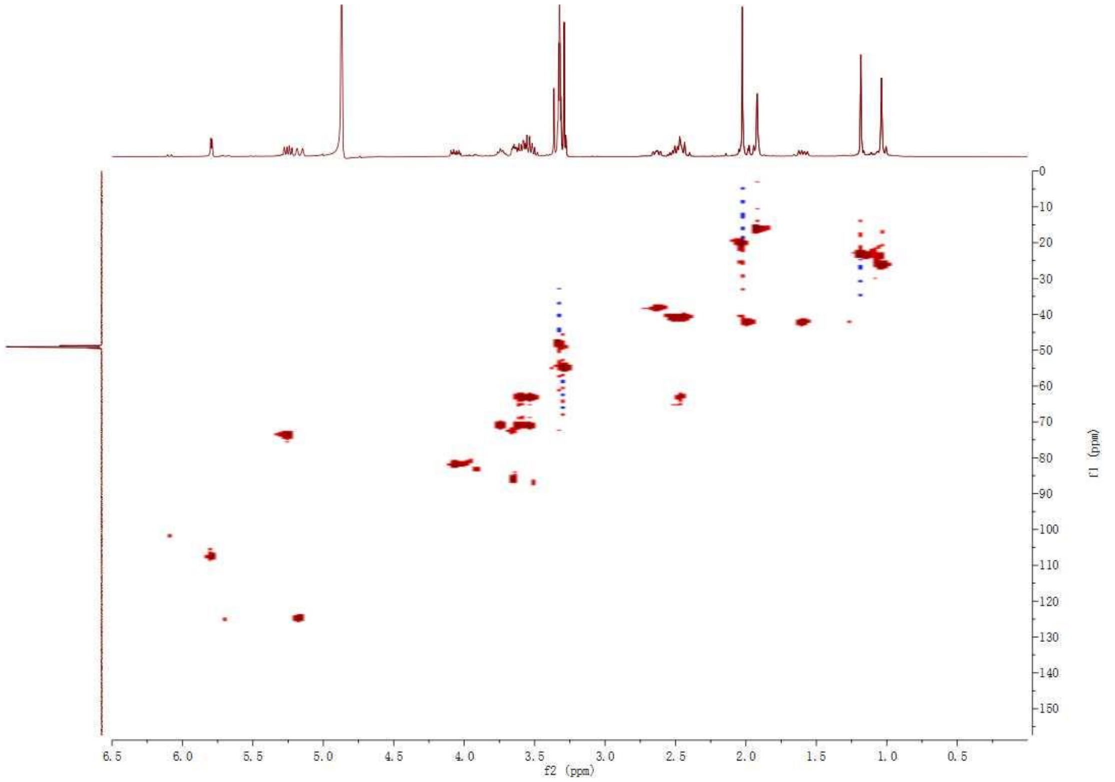

Figure S44. The HSQC (600 MHz, CD_3OD) spectrum of compound **6**.

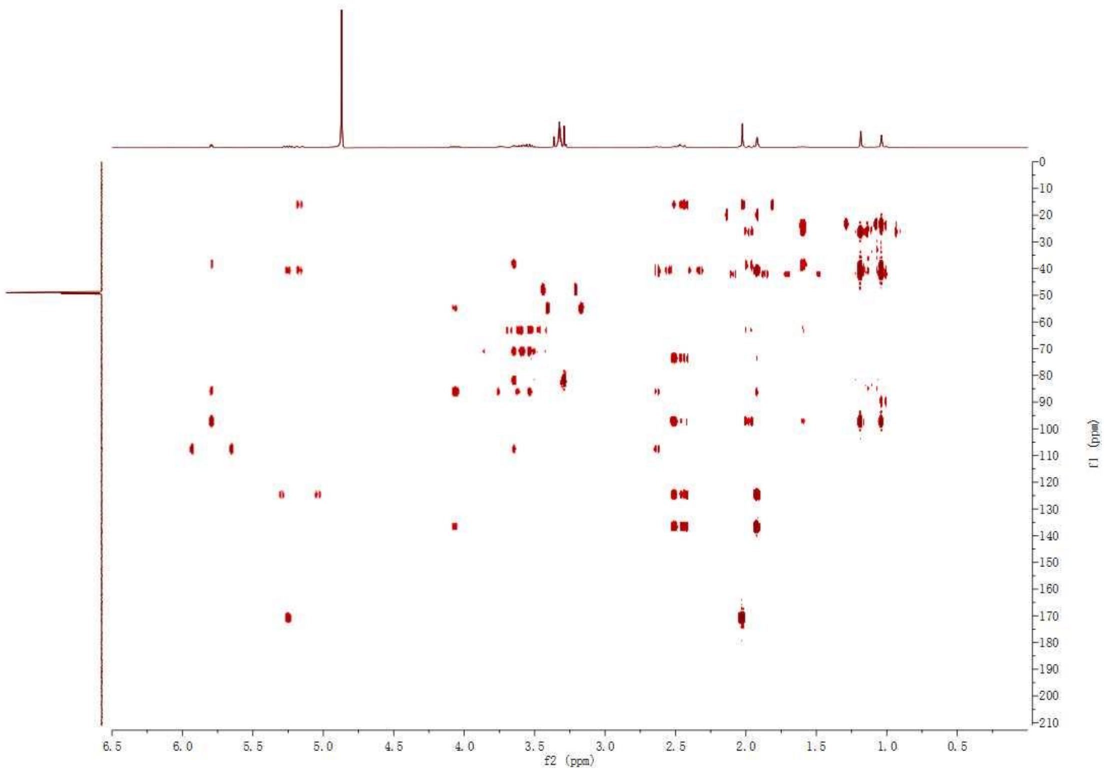

Figure S45. The HMBC (600 MHz, CD_3OD) spectrum of compound **6**.

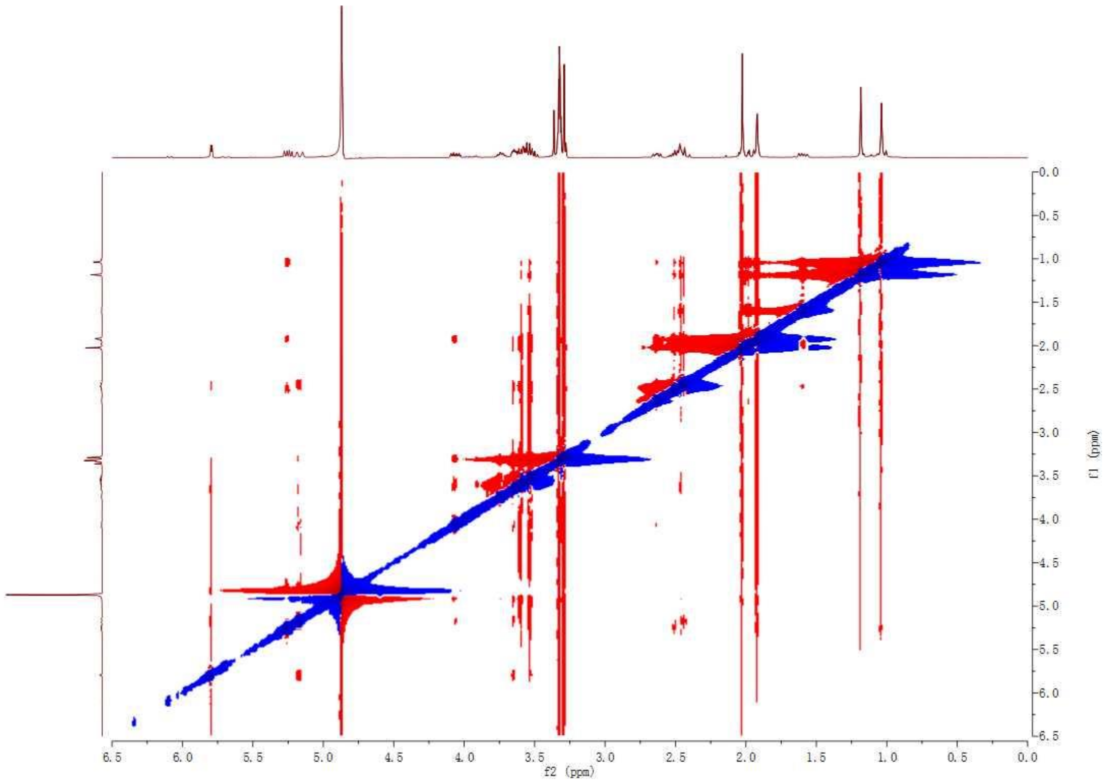

Figure S46. The ROESY (600 MHz, CD_3OD) spectrum of compound **6**.
